# Aromatic amino acid metabolism shapes autophagy-mediated adaptation to iron deprivation in glioblastoma cells

**DOI:** 10.1007/s10534-026-00809-7

**Published:** 2026-04-02

**Authors:** Kai Zhao, Lena Ludwig-Radtke, Yukai Wang, Tillmann Rusch, Christopher Nimsky, R. Verena Taudte, Jörg W. Bartsch

**Affiliations:** 1 Department of Neurosurgery, Marburg University, University Hospital Marburg, Baldingerstrasse, 35033 Marburg, Germany; 2 Metabolomics Core Facility, Marburg University, University Hospital Marburg, Baldingerstrasse, 35043 Marburg, Germany; 3 Research Group Translational Metabolomics, Marburg University, University Hospital Marburg, Baldingerstrasse, 35043 Marburg, Germany; 4 Department of Hematology, Oncology, and Immunology, Marburg University, University Hospital, Baldingerstrasse, 35043 Marburg, Germany; 5 Center for Mind, Brain and Behavior, CMBB Marburg, Hans-Meerwein-Strasse 6, 35032 Marburg, Germany

**Keywords:** Glioblastoma, Deferoxamine, Hypoxia, Autophagy, Apoptosis, Ferroptosis, Metabolomics, Aromatic amino acid

## Abstract

Glioblastoma (GBM) displays profound iron dependence and metabolic plasticity, yet how iron deprivation interfaces with stress-response pathways and amino acid metabolism in GBM remains incompletely understood. Deferoxamine (DFO), an iron chelator and hypoxia mimetic, is widely used experimentally, but the integration of autophagy, apoptosis, and ferroptosis under DFO-induced stress is unclear. This study aims to clarify how iron chelation reshapes stress signaling and metabolism in GBM cells and to define the role of aromatic amino acid metabolism in autophagy-mediated adaptation to iron deprivation. U87 and U251 human GBM cell lines were treated with DFO in the presence or absence of pan-caspase inhibitor Q-VD-OPh, the autophagy inhibitor CQ, or L-phenylalanine and L-tyrosine. AlamarBlue assessed cell viability. Hypoxia, autophagy, apoptosis, and ferroptosis-related genes and proteins were analyzed by qPCR, western blotting, and immunofluorescence. Global metabolic alterations were profiled by untargeted UHPLC–HRMS-based metabolomics followed by multivariate and pathway enrichment analyses. DFO stabilized HIF-1α, robustly induced hypoxia-related gene expression, and reduced GBM cell viability, with U251 cells being more sensitive than U87. DFO induced an autophagy response associated with ULK1 upregulation and reduced mTOR transcript levels, accompanied by increased LC3-II and changes in p62 levels. A lysosomal inhibition-based assay further supported increased autophagic flux, and meanwhile engaged apoptosis, particularly in U251, where Q-VD-OPh significantly rescued viability. In contrast, DFO induces an iron-starvation signature and maintains ferroptosis-associated antioxidant markers (GPX4/SLC7A11). CQ co-treatment potentiated DFO cytotoxicity in both lines, indicating a cytoprotective role of autophagy. Metabolomics revealed extensive DFO-induced reprogramming of amino acid and central carbon metabolism, with aromatic amino acid metabolism emerging as key pathway. DFO decreased intracellular L-phenylalanine and L-tyrosine in U87 but not U251 cells. Combined L-P + L-T supplementation attenuated DFO-induced autophagic responses and further enhanced DFO-mediated cytotoxicity. Iron chelation by DFO establishes a multifaceted stress state in glioblastoma cells, characterized by *hypoxia-like* transcription, cytoprotective autophagy, apoptosis, and ferroptosis resistance, coupled to profound amino acid-centric metabolic remodeling. Aromatic amino acid metabolism modulates autophagic responses and a determinant of GBM susceptibility to iron deprivation. Therefore, targeting aromatic amino acid metabolism to disable protective autophagy may enhance the therapeutic efficacy of iron-based strategies in GBM.

**Supplementary Information:**

The online version contains supplementary material available at 10.1007/s10534-026-00809-7.

## Background

Glioblastoma (GBM) is the most prevalent and most lethal primary malignant brain tumor in adults, characterized by diffuse infiltration, high proliferation rates, extensive intratumoral heterogeneity, and profound metabolic adaptability. GBM remains essentially unaffected by existing multimodal treatment strategies (Pouyan et al. 2025). Current therapy regimes, including maximal safe surgical resection followed by radiotherapy with adjuvant temozolomide, offer only limited survival benefit, and recurrence is virtually inevitable (Thakur et al. 2022). The median overall survival remains approximately 12 to 15 months, reflecting not only intrinsic therapy resistance but also the remarkable capacity for GBM metabolic pathways remodeling and stress-response mechanisms to support continued growth under hostile microenvironmental conditions (Pouyan et al. 2025; Thakur et al. 2022).

A critical but underexplored feature of GBM biology is its reliance on iron metabolism (Singh et al. 2025). The majority of cancer cells, especially GBM cells, maintain an enlarged labile iron pool to sustain essential cellular processes, including oxidative phosphorylation, activation of *de novo* nucleotide biosynthesis, chromatin remodeling, ROS detoxification, and activity of iron-dependent dioxygenases (Li et al. 2025; Shenoy and Connor 2023; Feng et al. 2023; Salazar-Ramiro et al. 2016). The rapid proliferation characteristic of GBM further amplifies iron demand, making iron availability a limiting factor for tumor progression (Caverzan and Ibarra 2024). Consequently, iron-dependent enzymes, including those in the mitochondrial respiratory chain and tricarboxylic acid (TCA) cycle, play central roles in GBM metabolic homeostasis (Liang et al. 2025). Recent studies have shown that GBM cells frequently upregulate transferrin receptor 1 (TFR1), downregulate ferroportin, and restructure iron uptake and storage systems to maximize intracellular iron retention (Caverzan and Ibarra 2024). These observations collectively highlight iron metabolism as both a fundamental feature of GBM pathophysiology and a potential metabolic vulnerability.

Deferoxamine (DFO), an FDA-approved chelator, originally used for iron overload disorders, is widely used as a hypoxia mimetic in experimental systems (Parker et al. 2023; Guo et al. 2005). DFO stabilizes hypoxia-inducible factor 1α (HIF-1α) by inhibiting prolyl hydroxylases, thereby inducing a transcriptional program associated with angiogenesis, metabolic restructuring, glycolytic activation, and cellular adaptation to low oxygen (Fan et al. 2014). Beyond HIF regulation, DFO exerts broad metabolic effects by depleting iron, an essential cofactor for many enzymatic systems, thereby disrupting iron-sulfur cluster biogenesis, mitochondrial respiration, redox homeostasis, DNA repair, lipid metabolism, and amino acid metabolism pathways (Ru et al. 2024). As a result, DFO can trigger a spectrum of cellular stress responses that include autophagy, apoptosis, ferroptosis suppression, and mitochondrial dysfunction (Jing et al. 2025; Dong et al. 2023). The dominance of these pathways is influenced by cell type, metabolic state, oxygen tension, and intrinsic antioxidant capacity. In GBM, where hypoxia signaling, redox adaptation, and metabolic rewiring are chronically engaged (Virtuoso et al. 2021), the manner in which iron deprivation interfaces with these pre-existing regulatory manners remains largely unresolved.

Despite growing interest in iron-targeted therapeutic strategies, several important mechanistic uncertainties limit our understanding of how GBM cells respond to DFO. First, the dominant cell-death pathways triggered by DFO remain unclear. Iron chelation can simultaneously influence multiple death programs, such as apoptosis via mitochondrial dysfunction, autophagy via mTOR-ULK1 signaling (Jing et al. 2025; Dong et al. 2023; Poole et al. 2021), and ferroptosis suppression through iron depletion, yet the relative contribution and interplay of these pathways in GBM remain unexplored. Whether these responses differ between GBM subtypes (such as U87 and U251) with distinct metabolic reprogramming is still unknown. Secondly, although iron availability is tightly linked to mitochondrial metabolism, amino acid metabolism, and antioxidant systems (Ward and Cloonan 2018), there are currently no comprehensive metabolomic investigations that examined how DFO remodels the metabolic patterns of GBM cells. In particular, iron-dependent enzymes regulate different biosynthetic and catabolic pathways, and their inhibition may lead to metabolic characteristics that determine cellular susceptibility to stress. Third, specific metabolic processes, especially those involving aromatic amino acids, have been implicated in modulating autophagy via metabolic stress-responsive pathways (Shin and Bang 2025; Nicklin et al. 2009). Whether such metabolic pathways serve as upstream determinants of adaptive autophagy and irreversible cytotoxicity in iron deprivation has not been addressed before. Addressing these issues is crucial for understanding the biological effects of iron chelation and for designing combination therapies that target metabolic and stress-response vulnerabilities.

To address these questions, we performed an integrated mechanistic and metabolomic analysis using two well-established GBM cell models, U87 and U251, which differ substantially in basal metabolic function, apoptotic sensitivity, and redox buffering capacity. We systematically examined how DFO influences hypoxia signaling, autophagy activation, apoptotic activation, ferroptosis-related markers, and overall cellular viability. Complementing these molecular and functional studies with untargeted metabolomics allowed us to map the global metabolic reorganization induced by iron deprivation and to identify metabolic pathways most strongly associated with the adaptive outcomes. Finally, based on the metabolomic identification of aromatic amino acids as DFO-responsive metabolites, we examined whether exogenous L-phenylalanine and L-tyrosine modify autophagy and alter cellular susceptibility to DFO, thereby revealing potential metabolic regulators of stress adaptation.

Collectively, this study provides an integrated perspective for understanding how iron chelation reshapes hypoxia-associated transcription, metabolic pathway, and the hierarchy of cell-death pathways in GBM. Moreover, our findings identify amino-acid metabolic pathway, which influence autophagic competence and determine GBM sensitivity to iron deprivation. These insights not only deepen the mechanistic understanding of iron metabolism in GBM, but also highlight the potential of therapeutic intervention based on combined targeting of iron homeostasis, metabolic regulation, and stress-adaptive pathways.

## Materials and methods

### Chemical reagents

All chemical reagents were freshly prepared before use. Deferoxamin (D9533, DFO) was purchased from Sigma-Aldrich. L-phenylalanine (HY-N0215) and L-tyrosine disodium salt (HY-N0473A) were purchased from MedChemExpress.

LC–MS grade methanol, water and acetonitrile (ACN) as well as ammonium formate (≥ 99%) and formic acid (LC–MS grad) were purchased from Th. Geyer GmbH & Co KG, Renningen and VWR, Darmstadt, Germany.

#### Cell culture

U251 human glioblastoma cells were obtained from ECACC (Cat Nr. #09063001; RRID:CVCL 0021). U87 cells were obtained from ATCC (HTB-14), corresponding to U87MG ATCC (RRID:CVCL_0022). This line is widely used as a GBM model but has been documented as a misidentified derivative that is not identical to the original Uppsala U-87MG line. All cells maintained under standard adherent culture conditions and were cultured in Dulbecco’s Modified Eagle Medium (DMEM, 41,965,039, Gibco, USA) supplemented with 10% fetal bovine serum (FBS, FBS-16A, Capricorn, Germany), 1% sodium pyruvate (12,539,059, Gibco, USA), 1% penicillin/streptomycin (FS-B, Capricorn, Germany), and 1% non-essential amino acids (NEAA, 12,084,947, Gibco, USA). All cells were incubated at 37 °C under 5% CO_2_ humidified incubator. L-phenylalanine (L-Phe, L-P) and L-tyrosine disodium salt (L-Tyr, L–T) were supplemented to standard DMEM (which already contains basal levels of these amino acids) at an additional concentration of 2 mM each. Stock solutions were prepared in sterile water, filter-sterilized (0.22 μm), and added to culture medium immediately before use. Medium pH was monitored and maintained at 7.2–7.4 after supplementation. Cells were regularly tested to be mycoplasma-free.

#### Cell viability assay

AlamarBlue HS cell viability reagent (A50101, ThermoFisher Scientific, USA) was used to assess cell viability of U87 and U251 cell lines. 4.0 × 10^3^ cells/well for 3d and 2.0 × 10^3^ cells/well for 5d were seeded in 96-well plate and incubated overnight. After treatment with different dose of DFO, cell viability for U87 and U251 were measured after 3 days or 5 days. Before measurement, 5μL of AlamarBlue reagent was added, incubated at least 1 h at 37 °C in the darkness. Fluorescence was measured with a Microplate Reader (FLUOstar OPTIMA Microplate Reader).

#### Caspase activity assay

Apoptosis was detected using the luminogenic caspase 3/7 substrate. Caspase-Glo® 3/7 (G8090, Promega, Heidelberg, Germany) was used for U87 and U251 cells. 4.0 × 10^3^ cells/well were seeded in 96 well plate and incubated overnight. After treatment with DFO, apoptosis of U87 and U251 were measured after 24 h. 20μL of Caspase reagent was added, mixed by shaking for 30 s, and incubated for 1 h at RT to avoid light. Luminescence was measured with a Microplate Reader luminometer (FLUOstar OPTIMA Microplate Reader).

#### RNA isolation and real time qPCR

5.0 × 10^5^ cells were seeded in 6-well plate overnight, and the indicated drugs applied for cells before RNA extraction. RNA was isolated by QIAzol reagent (79,306, Qiagen, Germany), OD260/280 ratio between 1.8 and 2.1 selected for the following reverse transcript. Afterwards, 2 μg RNA was subjected to the synthesis of cDNA by using RNA to cDNA EcoDry Premix (639,549, Takara, Kyoto, Japan) according to the manufacturer’s instructions. The RT-PCR reaction including 10μL GoTaq qPCR Master Mix (A6002, Pomega, Walldorf, Germany), 0.2 μl Supplemental CXR Reference Dye, 0.5μL primers, 7.3μL nuclease-free water, and 2μL cDNA. Initial denaturation at 95 °C for 2 min, then 40 amplification cycles including 95 °C for 15 s and 60 °C for 1 min. The primers for CA2, CA9, CA12, COX-1, COX-2, LC3, p62, ULK-1, BECN1, mTOR, AMPKa1, ATG4B, ATG5, ATG7, BAX, Casp3, Bcl-2, GPX4, TFR1, SLC7A11, XS-13 were purchased from Micosynth (Goettingen, Germany). The fold changes in gene expression relative to control were calculated by 2 ^– ΔΔCT^, all the relative expression data are shown as heat maps.

#### Protein isolation and western blot analysis

5.0 × 10^5^ cells were seeded in a 6-well plate overnight, cells treated with the indicated drugs for 24 h. Proteins were extracted with RIPA buffer including phenantrolin, protease inhibitor (A32955, Thermo Scientific, USA) and phosphatase inhibitor (A32957, Thermo Scientific, USA) after 3 times washing with ice-cold PBS. Subsequently, protein lysates were subjected to BCA (23,250, Thermo Scientific, USA) testing to obtain protein concentrations, 30 µg protein lysates were then boiled with sample reducing buffer (B0009, Invitrogen, USA) and Laemmli buffer for 5 min. Proteins were separated by 12.5% SDS polyacrylamide gel electrophoresis and transferred onto NC membranes (A29591442, GE Healthcare Life science, USA), and blocked with 5% non-fat milk (T145.3, Carl Roth GmbH, Karlsruhe, Germany) for 1 h at RT. All membranes were incubated overnight at 4 °C with the following primary antibodies, the following antibody were used: anti-PARP (1:1000 dilution, 5625, CST, Danvers, USA), anti-GPX4 (1:1000 dilution, 52,455, CST, Danvers, USA) in 5% BSA in TBST, and anti-p62 (2 μg/mL dilution, MAB8028, R&D Systems, Minneapolis, USA), anti-HIF-1 alpha (2 μg/mL dilution, AF1935, R&D Systems, Minneapolis, USA), anti-TRFR (1:5000 dilution, ab269513, Abcam, Cambridge, UK), anti-Casp3 (1:1000 dilution, 9661, CST, Danvers, USA), anti-MAP LC3 (1:500 dilution, sc-271625, Santa Cruz Biotechnology, Dallas, USA), and anti-β-Tubulin (1:2000 dilution, NB600-936, Novus Biologicals, Littleton, USA) in 5% milk in TBST. Subsequently, the NC membranes were incubated with secondary antibody Donkey (Dnk) pAb to Mouse (Ms) IgG (HRP) (ab97030, Abcam, Cambridge, UK) and Dnk pAb to Rabbit (Rb) IgG (HRP) (ab97064, Abcam, Cambridge, UK) in 5% milk in TBST or 5% BSA in TBSR for at least 1 h at RT. After 3 times TBST washing, the membranes detection was performed by utilizing theChemiDoc MP Imaging System (Bio-Rad Laboratories GmbH, Feldkirchen, Germany).

#### Metabolomics sample preparation

Cells were washed three times with pre-warmed PBS (37 °C) and put on dry ice. Cells were lysed and metabolites extracted by scraping the cells in 1 mL of pre-cooled (− 20 °C) 80% methanol. The extracts were transferred to 1.5 mL Eppendorf tubes and centrifuged at 16,000 rpm for 5 min at 4 °C. Supernatants were aliquoted (350 µL each) for reverse-phase (RP) chromatography and hydrophilic interaction liquid chromatography (HILIC), and an additional aliquot (250 µL) was set aside to prepare a pooled QC sample processed in parallel with all other samples. All aliquots were dried overnight in a speed vacuum concentrator and reconstituted in the appropriate RP or HILIC starting eluents prior to analysis.

#### Immunofluorescence staining

Cells were plated on coverslips pre-coated with collagen I (C7661, Sigma, Dreieich, Germany) in 24-well plates overnight. The following day, PBS was used to wash cells three times for 15 min at RT, fixed with 4% paraformaldehyde for 15 min at RT, and permeabilized with 0.3% Triton X-100 (T8787, Sigma, Dreieich, Germany) for 15 min at RT. Afterwards, 5% BSA blocking for 1 h. Cells were subsequently exposed to anti-MAP LC3 (1:250, sc-271625, Santa Cruz Biotechnology, Dallas, USA), anti-TRFR (1:50, ab269513, Abcam, Cambridge, UK), anti-Casp3 (1:400, 9661, CST, Danvers, USA) in 5% BSA at 4 °C overnight. The next day, after three times PBS washes, a donkey anti-mouse Alexa Fluor 488–conjugated secondary antibody (1:400, ab98794, Abcam, Cambridge, UK) was applied for at least 1 h at RT with protection from light. Nuclei were counterstained with Hoechst 33,342 for 15 min at RT in the dark. Finally, coverslips were mounted using an anti-fade medium prior to imaging.

#### Non-targeted metabolomics

All measurements were performed as described before (Krause et al. 2024) on a Vanquish UHPLC system coupled to an Orbitrap Exploris 480 mass spectrometer (Thermo Fisher Scientific, Dreieich, Germany) equipped with a heated electrospray ionization (HESI) source and controlled with Xcalibur software. Each sample was analyzed in both RP and HILIC modes, and data were acquired in positive and negative ionization. QC samples generated from pooled material were injected every five runs to monitor and correct for instrument drift (QC correction plots can be found in the Suppl. Figure S1). RP separations used an Acquity UPLC BEH C18 column (1.7 µm, 2.1 × 100 mm) with a matching guard column. Mobile phase A consisted of water with 0.1% formic acid, and mobile phase B of methanol with 0.1% formic acid. The gradient started at 0.5% B, increased to 98% B over 11 min, held until 15 min, and then returned to initial conditions by 20 min. HILIC runs employed an Acquity UPLC BEH Amide column (1.7 µm, 2.1 × 100 mm) and guard column. Mobile phase A was 10 mM ammonium formate with 0.1% formic acid; mobile phase B contained 10 mM ammonium formate in 95% ACN/5% water with 0.1% formic acid. The gradient began at 100% B, decreased to 50% B by 10 min, and returned to 100% B by 15.5 min.

For both chromatographic methods, the column temperature was consistently maintained at 40 °C, the flow rate was set at 0.35 mL/ min and the injection volume was 5 µL. The Orbitrap Exploris 480 was operated at 3.5 kV in positive mode and − 3 kV in negative mode. Vaporizer temperatures were 320 °C for HILIC and 250 °C for RP. The ion transfer tube temperature was 275 °C for all runs. Sheath, auxiliary, and sweep gases were set to 40, 8, and 1 a.u., respectively, using nitrogen 5.0. Spectra were acquired from m/z 70–800.

For MS1 scans, the resolution was 90,000, with standard AGC and maximum injection times of 100 ms (HILIC) or 384 ms (RP). In ddMS2 mode, resolution was set to 30,000 with standard AGC and an injection time of 54 ms.

Data were processed in Compound Discoverer 3.3 (Thermo Fisher Scientific), whereby the workflow for “untargeted metabolomics with statistics detect unknowns with ID using online databases and mzLogic” was applied. Alignment tolerances were set to 0.2 min for RP and 1 min for HILIC; mass tolerance was set to 5 ppm. Compound annotation was performed using mzCloud™, mzVault, ChemSpider, and mass lists (Thermo Fisher Scientific). After preprocessing including blank subtraction (maximum allowed ratio of sample vs blank to be considered as background was 5), features were manually reviewed by inspecting chromatograms and matching MS/MS spectra. Furthermore, metabolites likely occurring due to contamination were manually removed during the filtering process. Furthermore, incorrectly annotated features or features without annotation were labelled as unknowns.

Peak areas were normalized to cell counts (areas multiplied by 1/ cell count) and corrected using QC-based adjustments (Suppl. Figure S1). QC coverage was set to 50%, maximal QC area RSD was 30%, maximal corrected QC area RSD 25%. Peak lists from the four analytical modes (HILIC positive/negative and RP positive/negative) were merged in R. When the same compound appeared in more than one mode, the signal with the highest abundance was retained. The final merged dataset was used for downstream bioinformatic analysis. Peak annotations were categorized into four levels (see ID Quality column in Suppl. Table 1). ID Quality indicates metabolite annotation based on combined MS1 and MS2 data with a spectral matching score ≥ 80 compared to the respective online database (as specified in Suppl. Table 1). ID quality 2 refers to annotations derived from MS1 and MS2 data with a matching score ≥ 60. ID quality 3 includes annotations based on MS1 and MS2 data with a matching score ≤ 60. ID quality 4 denotes annotations based solely on MS1 data in the absence of supporting MS2 information.

#### Statistical analyses

Data are shown as the mean ± SD and analyzed using GraphPad Prism software (GraphPad Software Inc., San Diego, CA, USA). The IC_50_ values were determined by a non-linear regression method using the least-square fit. Unpaired Student’s t-tests were used for statistical comparison among two groups. Analysis of variance (ANOVA) was performed for multicomponent comparisons, applying Tukey’s post-hoc test. A p-value < 0.05 was considered as statistically significant. Further metabolomic data analyses i.e., pathway and enrichment analyses, were performed using MetaboAnalyst (version 6.0), an open online platform for comprehensive metabolomics data analysis. For this, only metabolites with ID quality 2 were used.

## Results

### Deferoxamine (DFO) stabilizes HIF-1α, induces hypoxia-related gene expression, and suppresses GBM cell viability

**Fig. 1:**
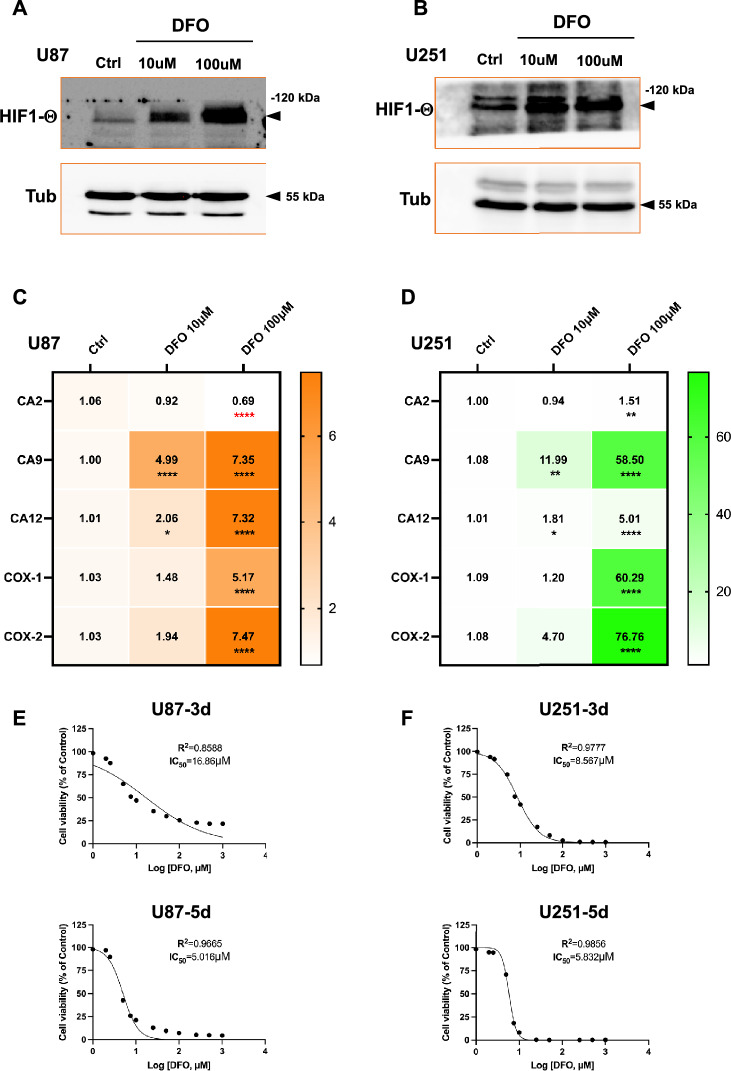
DFO stabilizes HIF1-α, induce *hypoxia-related* gene expression, and decreases viability in glioblastoma cells. **A**, **B** HIF1-α protein expression levels in U87 (**A**) and U251 (**B**) cells treated with deferoxamine (DFO, 10 μM and 100 μM) for 24 h, detected by western blot. As loading control, β-tubulin was used. **C**, **D** mRNA expression of *hypoxia-related* gene, CA2, CA9, CA12, COX-1, COX-2 in U87 (**C**) and U251 (**D**) cells following DFO treatment were detected via qPCR. Expression levels were normalized to control and displayed as fold changes in heatmaps. DFO markedly increased CA9, CA12, COX-1, and COX-2 expression in both cell lines. U87 **E** and U251 **F** cells treated with different doses of DFO for 3 and 5 days were used for cell viability by Alamar blue. Dose–response curves are shown with calculated IC_50_ values (U87: 16.86 μM at 3d, 5.016 μM at 5d; U251: 8.567 μM at 3d, 5.832 μM at 5d), indicating higher sensitivity of U251 cells to DFO, especially at day 3. All data are presented from three independent experiments. Statistical significance was determined by one-way ANOVA with post hoc test (*p < 0.05, **p < 0.01, ****p < 0.0001)

To investigate the effect of iron chelation on hypoxia-related signaling in glioblastoma, U87 and U251 cells were exposed to increasing concentrations of DFO (10 μM and 100 μM). As shown in Fig. 1A-B, DFO treatment markedly enhanced HIF-1α protein accumulation, confirming stabilization of HIF-1α under iron-depleted conditions. Consistent with this, DFO induced robust transcriptional activation of HIF-1 responsive genes, with distinct expression patterns between U87 and U251 lines. In U87 cells (Fig. 1C), DFO significantly upregulated CA9, CA12, COX-1, and COX-2 mRNA expression (up to ~ sevenfold increase at 100 μM), whereas CA2 was modestly downregulated. Conversely, U251 cells (Fig. 1D) exhibited a far more pronounced transcriptional response, particularly for CA9, COX-1, and COX-2, which increased over 50–70 fold increase following 100 μM DFO exposure, indicating an amplified HIF-1 induced metabolic adaptation. Functionally, DFO suppressed cell viability in a concentration and time dependent manner.

(Fig. 1E, F). The calculated IC_50_ values were 16.86 μM and 5.02 μM in U87 cells after 3 and 5 days of treatment, respectively, whereas U251 cells displayed greater sensitivity with IC_50_ values of 8.57 μM and 5.83 μM. Taken together, these findings demonstrate that DFO promotes HIF-1α stabilization, triggers *hypoxia-related* gene activation, and reduces GBM cell viability, with U251 cells exhibiting heightened transcriptional responsiveness and chemosensitivity compared with U87 cells.

### DFO induces autophagy-associated responses in GBM cells

**Fig. 2:**
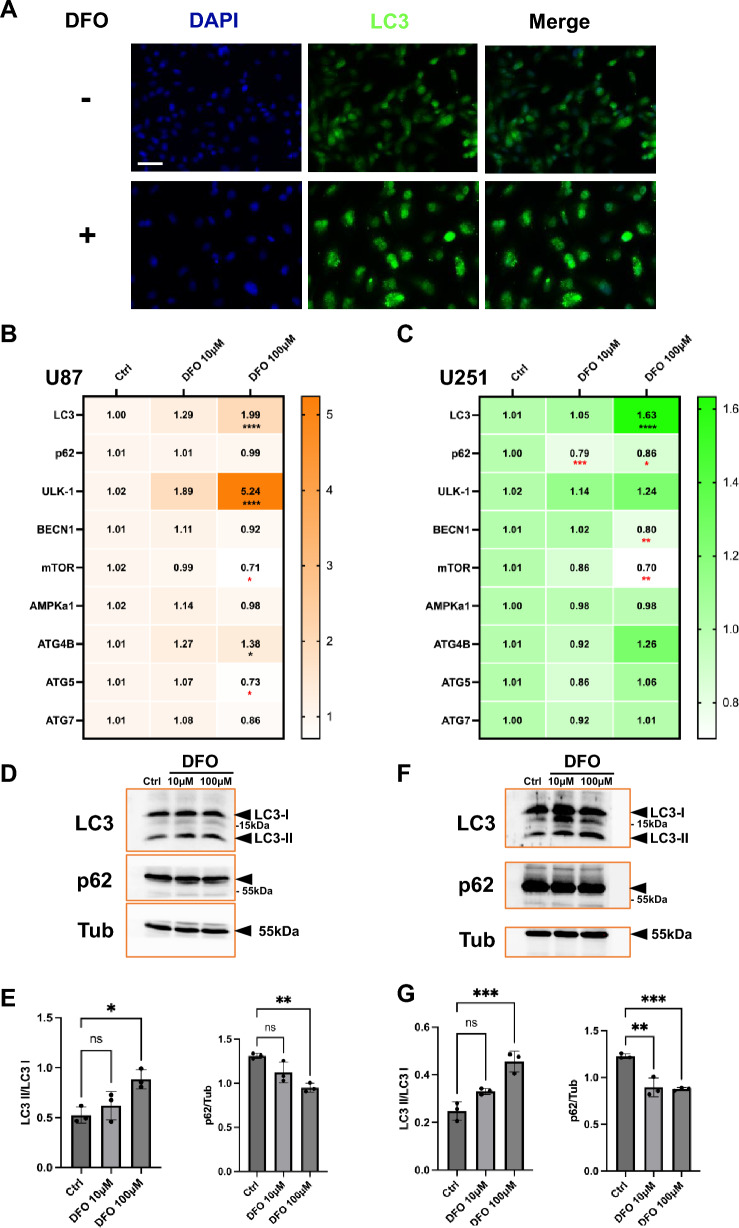
DFO induces autophagy in U87 and U251 cell lines. **A** Immunofluorescence staining of LC3 in U87 cells treated with or without DFO (100 μM, 24 h). DAPI was used for nuclear staining. Enhanced punctate LC3 signal was observed in DFO-treated cells, indicating increased autophagosome formation. Scale bar in A, 40 µm; **B**, **C** Quantitative real-time PCR analysis of autophagy-related gene expression in U87 (**B**) and U251 (**C**) cells following DFO treatment (10 μM and 100 μM, 24 h), including LC3, p62, ULK1, BECN1, mTOR, AMPKα1, ATG4B, ATG5, and ATG7. Fold changes were normalized to control cells and are presented as heatmaps. **D**–**G** Western blot analysis of LC3 and p62 protein expression in U87 (**D**) and U251 (**F**) cells treated with DFO (10 μM and 100 μM, 24 h). Quantification of LC3-II/LC3-I ratio and p62 expression (**E**, **G**) confirmed DFO significantly increased LC3-II accumulation while reducing p62 levels, consistent with autophagy activation. Data are presented as mean ± SD from three independent experiments. Statistical significance was determined using one-way ANOVA with post hoc testing (*p < 0.05, **p < 0.01, ***p < 0.001)

To investigate whether DFO regulates autophagy in GBM cells, LC3 immunofluorescence was performed in U87 cells, DFO treatment (100 μM, 24 h) markedly increased the intensity of LC3-positive puncta, reflecting enhanced autophagosome formation (Fig. 2A). qPCR analysis showed transcriptional activation of autophagy-related genes following DFO exposure (Fig. 2B). Among these, ULK1 was the most significantly upregulated gene (1.89 to 5.24 fold change), accompanied by modest increases in LC3 and ATG4B expression and a notable downregulation of mTOR transcripts (to approximately 0.71 fold change at 100 μM), suggesting involvement of the ULK1–mTOR regulatory node in the autophagy response. Expression of BECN1, AMPKα1, ATG7, and p62/SQSTM1 remained largely unchanged, suggesting that DFO primarily influences autophagy initiation rather than upstream complex formation. Afterwards, western blot confirmed that DFO treatment dose-dependently increased the conversion of LC3-I to LC3-II (Fig. 2D). Quantification revealed a significant elevation of the LC3-II/LC3-I ratio, particularly at 100 μM DFO (Fig. 2E), changes in p62 protein levels were observed (Fig. 2E), however, p62 turnover can be regulated at multiple levels, its reduction alone was not interpreted as definitive evidence of increased autophagic flux. These findings indicate that iron chelation triggers autophagosome formation and is associated with enhanced autophagic activity in U87 cells. In U251 cells, a similar but more pronounced autophagic response was observed. DFO treatment upregulated ULK1 and LC3 transcripts while downregulating mTOR and p62 mRNA levels (Fig. 2C). Consistent with transcriptional changes, western blot revealed strong accumulation of LC3-II and a significant increase in the LC3-II/LC3-I ratio at 100 μM DFO (Fig. 2F, G). Meanwhile, p62 protein levels were significantly reduced (Fig. 2F, G), further supporting increased autophagic activity.

To more directly assess autophagic flux, we performed a lysosomal inhibition-based assay using chloroquine (CQ). At 24 h, DFO treatment increased LC3-II levels in both U87 and U251 cells. Importantly, CQ co-treatment resulted in a further accumulation of LC3-II compared with DFO alone (Suppl. Figure S2), consistent with increased autophagic flux rather than impaired autophagosome degradation. These findings indicate that iron chelation activates autophagy and is associated with enhanced autophagic flux in GBM cells.

Collectively, these results demonstrate that DFO robustly activates autophagy in both U87 and U251 GBM cell lines, accompanied by ULK1 upregulation and reduced mTOR transcript levels, leading to increased LC3 lipidation and alterations in p62. The coordinated increase in LC3-II together with CQ-dependent LC3-II accumulation and p62 alterations support that DFO induces autophagy and is associated with increased autophagic flux. Notably, U251 cells exhibited a slightly stronger response in western blot, suggesting greater sensitivity to iron chelation-induced metabolic stress.

### DFO promotes apoptosis in GBM cells

**Fig. 3:**
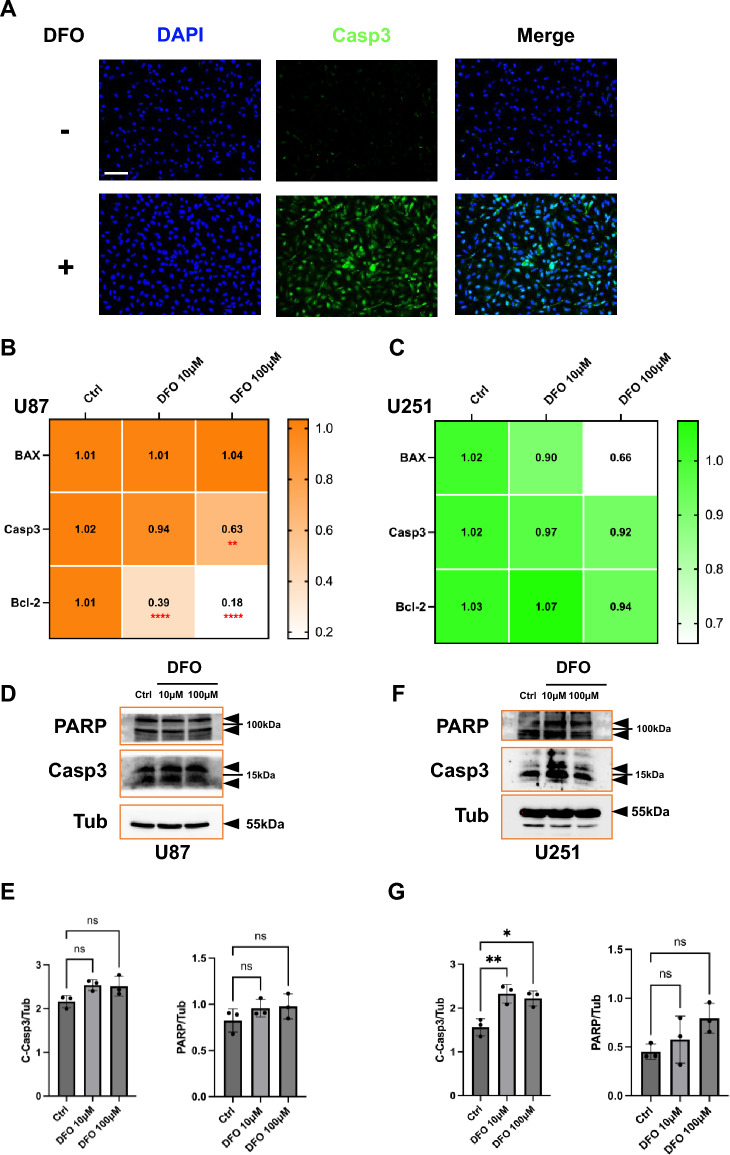
Effects of DFO on apoptosis in U87 and U251 glioblastoma cells. **A** Immunofluorescence staining of Casp3 in U87 cells treated with or without DFO (100 μM, 24 h). DAPI was used for nuclear counterstaining. Enhanced Casp3 signal was observed after DFO treatment. Scale bar in A, 80 µm **B**, **C** qPCR analysis of apoptosis-related genes (BAX, Casp3, Bcl-2) in U87 (**B**) and U251 (**C**) cells treated with DFO (10 μM and 100 μM, 24 h). Heatmaps represent fold changes normalized to untreated controls. **D**–**G** Western blot analysis of cleaved PARP (C-PARP) and cleaved caspase-3 (C-Casp3) in U87 (**D**) and U251 (**F**) cells following DFO treatment. Quantification of C-Casp3 and PARP expression in U87 (**E**) and U251 (**G**) normalized to Tubulin as shown. Data are presented as mean ± SD from at three independent experiments. Statistical analysis was performed using one-way ANOVA with post hoc testing (*p < 0.05, **p < 0.01, ns = not significant)

To determine whether DFO induces apoptosis in GBM cells, we examined caspase-3 activation and apoptosis-related signaling in both U87 and U251 cells. In U87 cells, caspase-3 immunofluorescence revealed a marked increase in cytoplasmic Casp3 signal after 24 h of DFO treatment, indicating activation of the apoptotic machinery (Fig. 3A).

qPCR analysis demonstrated that DFO significantly downregulated the anti-apoptotic gene Bcl-2, while BAX expression remained unchanged and Casp3 mRNA showed a mild decrease (Fig. 3B). Furthermore, western blot analysis showed that DFO treatment increased in cleaved caspase3 (Fig. 3D, E). Consistently, Caspase-Glo assays revealed a corresponding increase in caspase activity (Suppl. Figure S3). PARP cleavage did not show a significant change (Fig. 3E, right). Collectively, these results suggest that DFO promotes U87 cells for apoptosis through suppression of Bcl-2 and early activation of caspase-3. In U251 cells, DFO treatment elicited a more pronounced apoptotic response. At the transcriptional level, BAX expression decreased slightly, while Bcl-2 and Casp3 remained relatively stable (Fig. 3C). In contrast, western blotting revealed strong caspase-3 activation, with a significant increase in cleaved caspase-3 at 100 μM DFO (Fig. 3F, G), whereas PARP levels remained largely unchanged (Fig. 3G).

### DFO induces an iron-starvation signature and maintains GPX4 / SLC7A11 expression

**Fig. 4:**
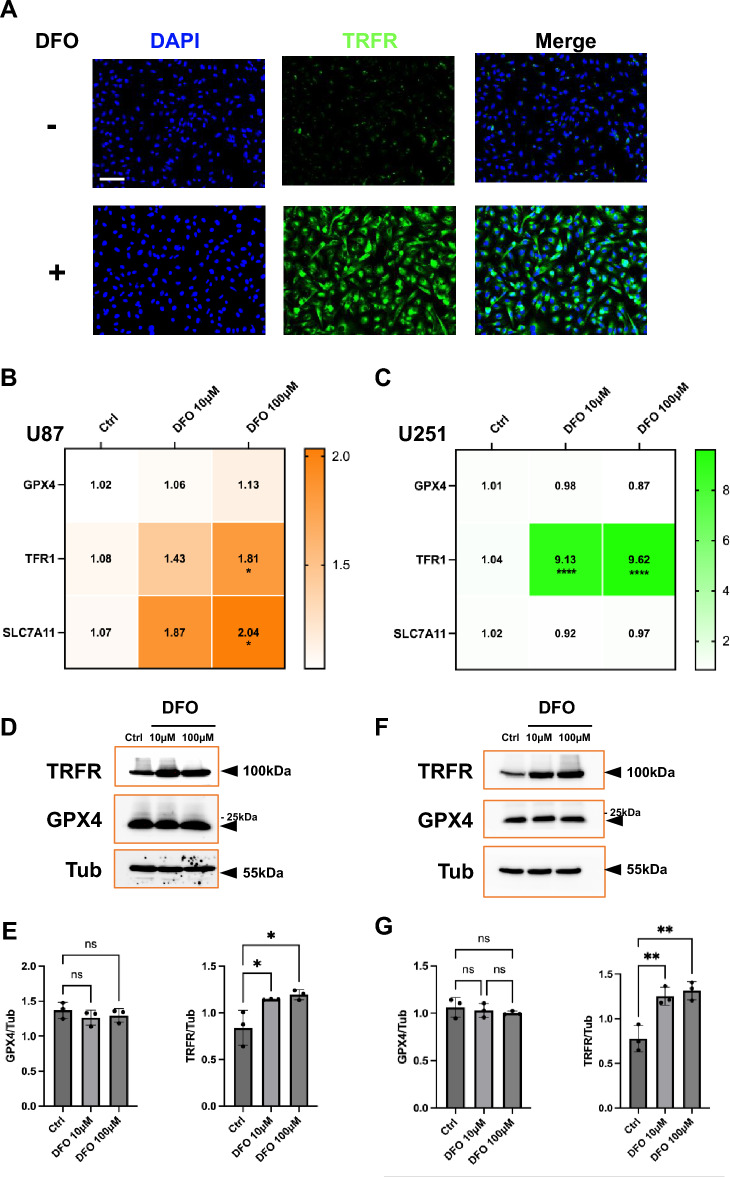
DFO regulates ferroptosis-associated gene and protein expression in U87 and U251 cells. **A** Immunofluorescence staining of TRFR in U87 cells treated with or without DFO (100 μM, 24 h). Nuclei were counterstained with DAPI. DFO treatment markedly enhanced TRFR signal intensity, indicating increased iron uptake capacity. Scale bar in A, 80 µm; (B-C) qPCR analysis of ferroptosis-related genes (GPX4, TFR1, SLC7A11) in U87 **B** and U251 **C** cells following exposure to DFO (10 μM and 100 μM, 24 h). Heatmaps represent mean fold changes relative to controls. **D**–**G** Western blot analysis of TRFR and GPX4 protein expression in U87 (**D**) and U251 (**F**) cells treated with DFO (10 μM and 100 μM, 24 h). Quantification of GPX4 and TRFR expression in U87 (**E**) and U251 (**G**) normalized to Tubulin as shown. Data are presented as mean ± SD from three independent experiments. Statistical analysis was performed using one-way ANOVA with post hoc testing (*p < 0.05, **p < 0.01, ns = not significant)

To assess whether iron chelation antagonizes ferroptosis in GBM, we examined transferrin receptor (TFR1/TRFR), GPX4, and SLC7A11 in both U87 and U251 cells. In U87 cells, immunofluorescence showed a clear increase in TRFR staining after 24 h DFO treatment, indicating a compensatory response to intracellular iron depletion (Fig. 4A).

Consistently, qPCR revealed upregulation of TFR1 together with an induction of the cystine/glutamate antiporter SLC7A11, while GPX4 mRNA expression remained unchanged (Fig. 4B). Meanwhile, western blot demonstrated a significant accumulation of TRFR, whereas GPX4 protein remained unchanged (Fig. 4D, E). In U251 cells, ferroptosis was restrained as well, though distinct transcriptional patterns. TFR1 mRNA increased strikingly, while SLC7A11 remained near baseline and GPX4 transcripts decreased slightly (Fig. 4C). Despite this modest reduction at the mRNA level, GPX4 protein expression was maintained, and TRFR protein was significantly induced with DFO (Fig. 4F, G). Taken together, in both U87 and U251 cells, DFO induces an iron-starvation signature (TRFR/TFR1 upregulation) without loss of GPX4 and, in U87 cells, with reinforcement of SLC7A11. These changes are not consistent with canonical ferroptotic features and instead reflect an iron-starvation signature accompanied by preserved GPX4/SLC7A11 expression in U87 and U251 GBM cell lines.

### Pharmacological inhibition of apoptosis or autophagy reveals distinct roles in DFO-induced cytotoxicity

**Fig. 5:**
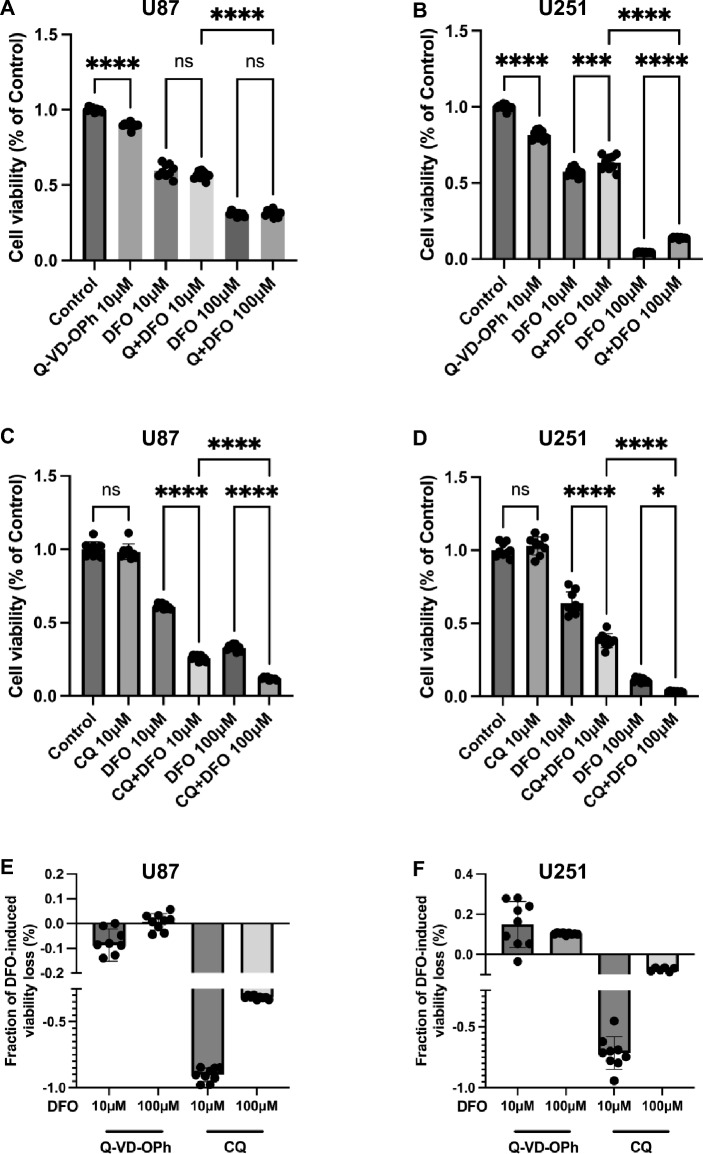
Effects of DFO in combination with apoptosis or autophagy inhibitors on cell viability in U87 and U251 cells. **A**, **B** Cell viabilities of U87 (**A**) and U251 (**B**) cells treated with DFO (10 μM or 100 μM, 3d) in the presence or absence of the pan-caspase inhibitor Q-VD-OPh (10 μM). **C**, **D** Cell viabilities of U87 (**C**) and U251 (**D**) cells treated with DFO (10 μM or 100 μM, 3d) in the presence or absence of the autophagy inhibitor chloroquine (CQ, 10 μM). **E**, **F** Relative contribution to DFO-induced cell death in U87 (**E**) and U251 (**F**) cells. Positive values indicate partial rescue; negative values indicate exacerbation of DFO-induced cytotoxicity, Fraction = (DFO + inhibitor − DFO) / (Ctrl − DFO). Data are presented as mean ± SD from three independent experiments (n = 3). Statistical analysis was performed using one-way ANOVA with post hoc testing (*p < 0.05, ***p < 0.001, ****p < 0.0001, ns = not significant)

To clarify the contribution of apoptosis and autophagy to DFO-mediated cell death, U87 and U251 GBM cells were co-treated with the pan-caspase inhibitor Q-VD-OPh (Fig. 5A, B) or the lysosomal/autophagy inhibitor chloroquine (CQ) (Fig. 5C, D), followed by Alamar Blue viability.

In U87 cells, DFO significantly reduced cell viability in a dose-dependent manner (Fig. 5A). Co-treatment with Q-VD-OPh resulted in a mild but non-significant change in viability compared with DFO alone, suggesting that apoptotic mechanisms play only a minor role in DFO-induced cell death in U87 cell. In contrast, in U251 cells, Q-VD-OPh markedly reversed the cytotoxic effect of DFO at both 10 μM and 100 μM compared with DFO alone incubation (Fig. 5B). This rescue demonstrates that DFO induced cell death in U251 cells is largely caspase-dependent, consistent with activation of apoptotic signaling pathways observed before. To further examine the involvement of autophagy, both cell lines were treated with DFO in the presence or absence of CQ (Fig. 5C, D). CQ alone had minimal impact on viability; however, CQ co-administration with DFO significantly enhanced cytotoxicity in both U87 and U251 cells compared with DFO alone. The exacerbate of DFO-induced cytotoxicity by CQ suggests that autophagy exerts a cytoprotective role as an adaptive mechanism that alleviates iron chelation-induced stress responses.

All in all, these results indicate that DFO triggers distinct cell-death modalities in GBM cells, apoptosis contributes substantially to DFO-induced cytotoxicity, as evidenced by Q-VD-OPh mediated rescue, especially in U251 cells. In both U87 and U251 lines, autophagy inhibition sensitizes cells to DFO, underscoring the protective role of autophagy in maintaining cell survival under iron-deficient conditions.

To quantitatively compare the relative impact of apoptosis inhibition and autophagy inhibition on DFO-induced viability loss, we calculated the fraction of DFO-induced viability loss modified by each inhibitor (Fraction = (DFO + inhibitor—DFO) / (Ctrl—DFO); Fig. 5E, F). Positive values indicate partial rescue, whereas negative values indicate exacerbation. Q-VD-OPh produced positive fractions that were more pronounced in U251 cells, whereas CQ consistently showed negative fractions in both cell lines. Collectively, these results indicate that apoptosis contributes more prominently to DFO-induced cytotoxicity in U251 cells, while intact autophagy function mitigates DFO-induced stress and supports cell survival in both GBM cells.

### DFO induces extensive metabolic reprogramming in U87 and U251 cell lines, primarily affecting amino acid and central carbon metabolism

**Fig. 6:**
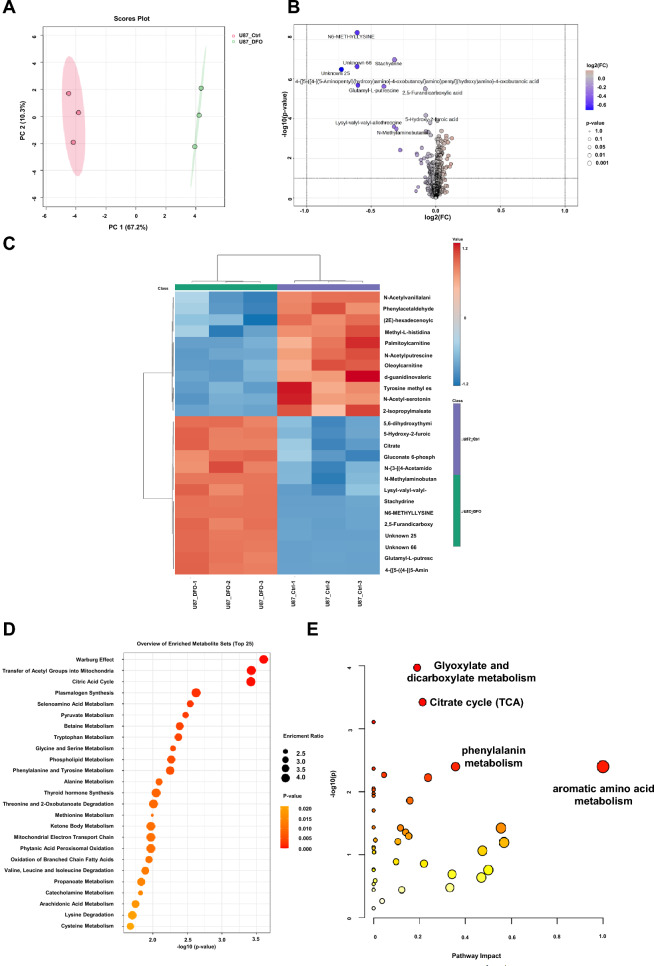
Metabolomic profiling of U87 cells in response to DFO treatment. Results of metaboAnalyst are demonstrated as **A** PCA plot the horizontal coordinates are the first principal component PC1, the vertical coordinates are the second principal component PC2, and the ellipse is the 95% confidence interval. Different groups are labeled with different colors (Pink for the control group and Green for the DFO group), indicating distinct metabolic alterations. **B** Volcano plot of differential metabolites shown as the log2 (fold change) versus the -log10 (adjusted p-value). **C** Heatmap of hierarchical clustering of significantly altered metabolites, demonstrating different metabolic signatures between control and DFO-treated U87 cells. KEGG pathway enrichment analysis of the top 25 enriched pathways shown as in (**D**), (**E**) Pathway analysis, the x-axis indicates pathway impact values, and the y-axis represents − log10 (p) values from enrichment analysis. Each dot corresponds to a metabolic pathway, with dot size proportional to the impact value and dot color reflecting statistical significance. The color gradient from yellow to red indicates decreasing p values. Data collections from three independent experiments (n = 3)

**Fig. 7:**
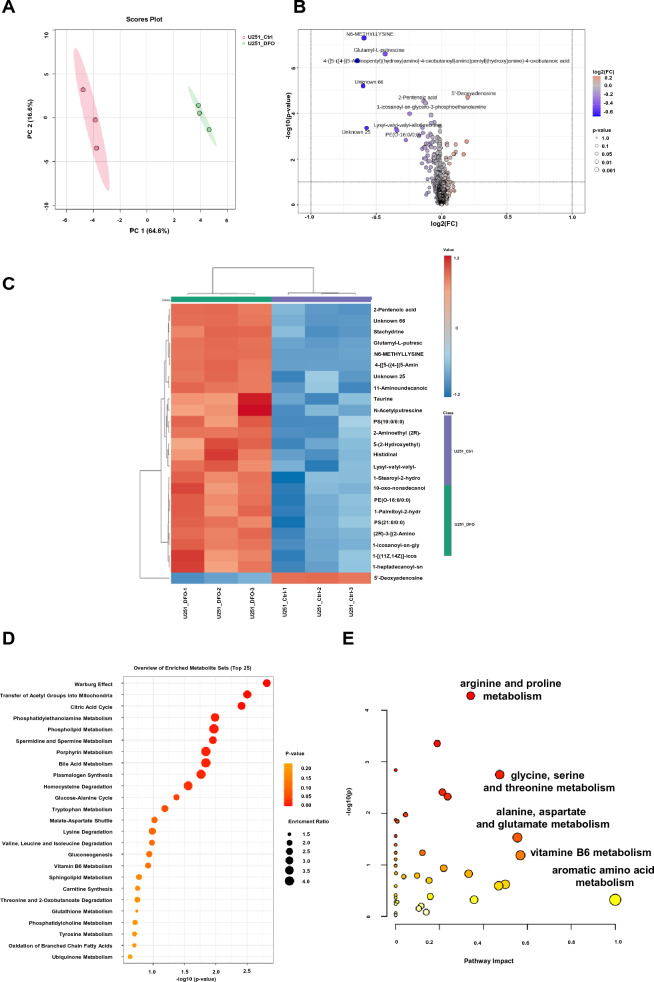
Metabolomic profiling of U251 cells in response to DFO treatment. Analyses were performed as described in Fig. 6. **A** PCA plot, **B** volcano plot, **C** hierarchical clustering heatmap of significantly altered metabolites. **D** KEGG pathway enrichment analysis, and **E** pathway analysis. Data are shown for U251 cells treated with or without DFO. Data collections from three independent experiments (n = 3)

Metabolomic profiling coupled with multi-variate and pathway analyses was performed to investigate the metabolic alterations induced by DFO in U87 and U251 cells. Principal component analysis (PCA) showed clear separation between control and DFO-treated groups in both cell lines, with clustered replicates within each group, reflecting treatment-dependent metabolic reprogramming (Figs. 6A and 7A).

Volcano plot analyses identified significantly altered metabolites following DFO exposure (Figs. 6B, 7B, DFO as positive control). Among the most prominent changes were increases in several amino acids and their derivatives, including N^6^-methyl-lysine, proline betaine, Glutamyl-L-putrescine, Lysyl-valyl-valyl-allothreonine. Hierarchical clustering analysis demonstrated a distinct metabolic profile for the DFO-treated groups, characterized by coordinated shifts in amino acids and organic acids (Figs. 6C and 7C). Pathway enrichment and topology analyses further highlighted the metabolic modules most impacted by DFO treatment (Figs. 6D, E, and 7D, E). In both U87 and U251 cells, DFO significantly changed the abundance of compounds present in multiple amino acid pathways, including aromatic amino acid metabolism, valine/leucine/isoleucine metabolism, arginine and proline metabolism, phenylalanine/tyrosine/tryptophan metabolism, alongside alterations in the tricarboxylic acid (TCA) cycle. Notably, in U251 cells, glutathione metabolism also emerged as a highly enriched, suggesting enhanced redox regulation in response to iron chelation stress. Collectively, these data demonstrate that DFO induces an amino acid-centric metabolic remodeling in GBM cells. While both U87 and U251 exhibit strong metabolic responses to iron depletion, the U251 metabolome displays a more pronounced activation of glutathione-associated antioxidant responses, consistent with an induced adaptive metabolic response to DFO-induced oxidative and iron-limiting conditions.

### DFO distinctly modulates phenylalanine/tyrosine/tryptophan metabolism between U87 and U251 GBM cells

**Fig. 8:**
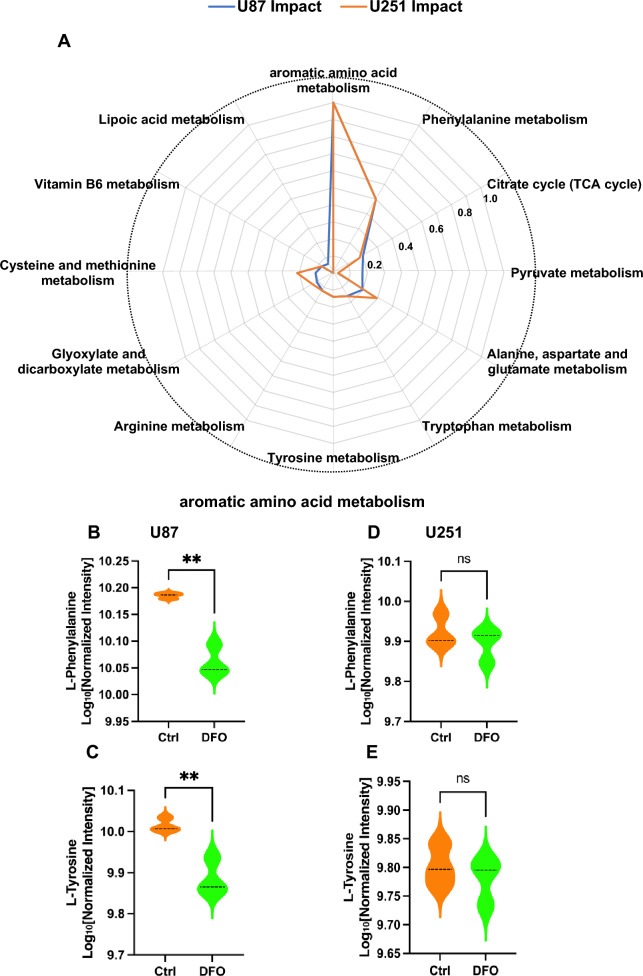
Comparison of phenylalanine/tyrosine/tryptophan metabolism pathways in U87 and U251 cells following DFO treatment. **A** Radar plot of pathway impact analysis comparing U87 and U251 cells under DFO treatment, indicating differential responses in amino acid metabolism, particularly phenylalanine/tyrosine/tryptophan metabolism. **B**, **C** Violin plots showing normalized intensities (log10 scale) of L-phenylalanine (**B**) and L-tyrosine (**C**) in U87 cells. **D**, **E** Violin plots showing normalized intensities of L-phenylalanine (**D**) and L-tyrosine (**E**) in U251 cells. Data are presented as mean ± SD from independent experiments. Statistical significance was assessed using Student’s t-test (**p < 0.01; ns, not significant)

To further explore the metabolic pathways most affected by DFO treatment, comparative pathway impact analysis was performed to directly contrast DFO-induced metabolic responses between U87 and U251 cells. The radar plot (Fig. 8A) revealed that the phenylalanine/tyrosine/tryptophan metabolism the highest impact score in both cell lines, but with markedly greater enrichment in U87 cells relative to U251. Other pathways, such as phenylalanine metabolism, TCA cycle, and alanine, aspartate, and glutamate metabolism, showed moderate contributions, indicating that DFO induces both amino acid and core metabolic remodeling, with cell line-specific differences. Given the prominence of aromatic amino acid metabolism in pathway enrichment, the intracellular levels of L-phenylalanine and L-tyrosine were examined in U87 and U251 cells. In U87 cells, DFO treatment significantly decreased both metabolites compared with the control group (Figs. 8B, C), suggesting aromatic amino acid metabolism is overutilized or downregulated under iron chelation. In contrast, U251 cells show no statistically significant changes in either L-phenylalanine or L-tyrosine following DFO exposure (Figs. 8D, E), despite global enrichment of related pathways in the network analysis. Together, these results demonstrate that DFO perturbs phenylalanine, phenylalanine/tyrosine/tryptophan metabolism, depending on different cell types, with U87 cells showing pronounced reductions in aromatic amino acid abundance, while U251 cells maintain relative metabolic stability. This difference suggests that U87 cells undergo stronger metabolic remodeling in amino acid metabolism in response to iron deprivation, whereas U251 cells maintain the stability of aromatic amino acids, potentially reflecting enhanced metabolic compensation capacity under DFO-induced stress.

### Combined treatment with L-phenylalanine and L-tyrosine enhances DFO-induced cytotoxicity by and attenuates autophagy-associated marker changes in U87 and U251 GBM cells

**Fig. 9:**
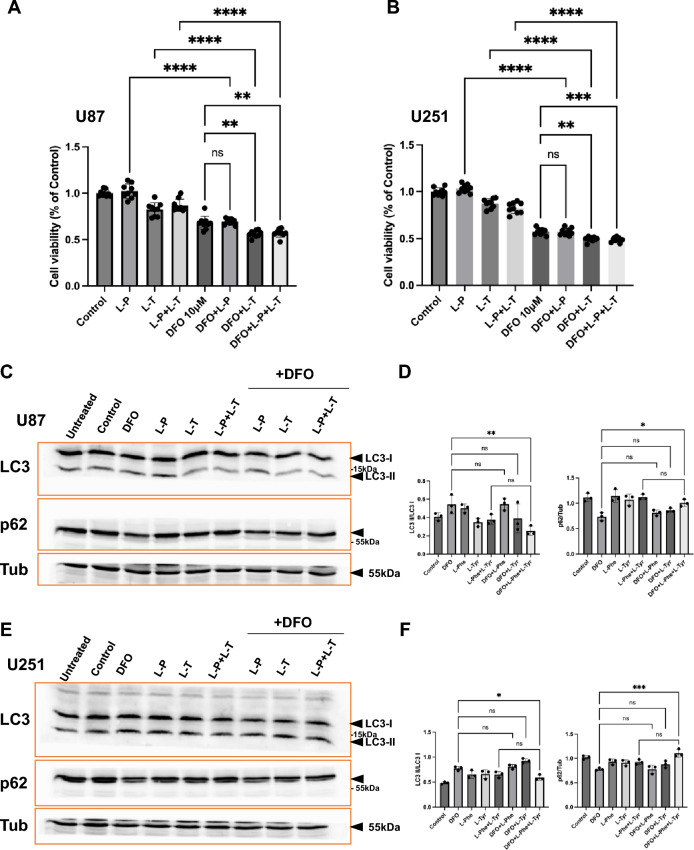
Effects of L-phenylalanine (L-P) and L-tyrosine (L–T) on cell viability and autophagy markers in U87 and U251 cells under DFO treatment. **A**, **B** Cell viability measured by Alamar Blue assay in U87 (A) and U251 (B) cells treated with 2 mM L-P, 2 mM L–T, or their combination, in the presence or absence of DFO (10 μM, 24 h). Both amino acids further reduced cell viability in DFO-treated cells compared with controls. **C**, **D** Western blot analysis of LC3 and p62 expression in U87 cells (**C**). Quantification show changes in LC3-II/LC3-I ratio and p62 levels (**D**). L-P and L–T co-treatment with DFO further enhanced autophagy activation compared with single treatments. **E**, **F** Western blot analysis of LC3 and p62 in U251 cells (**E**), and quantification show that L-P and L–T similarly modulated LC3-II/LC3-I ratio and p62 expression (**F**) in the presence of DFO. Tubulin (“Tub”) was used as a loading control. Data are presented as mean ± SD from at least three independent experiments. Statistical analysis was performed using one-way ANOVA with post hoc testing (*p < 0.05, **p < 0.01, ***p < 0.001, ****p < 0.0001, ns = not significant)

To further elucidate the relationship between aromatic amino acid metabolism and DFO-induced cell death, we examined the effects of L-phenylalanine (L-P) and L-tyrosine (L–T) supplementation on cell viability and autophagic activity in U87 and U251 GBM cells. Alamar Blue assays revealed that DFO treatment (10 μM, 24 h) significantly reduced cell viability in both cell lines, consistent with our previous findings (Figs. 9A, B). Supplementation with either L-P or L-T alone had minimal effects, whereas combined treatment with L-P + L-T moderately decreased viability compared to the respective controls. Notably, co-treatment with DFO and L-P + L–T further potentiated cytotoxicity, leading to significantly lower viability than DFO treatment alone (Figs. 9A, B). These data indicate that exogenous aromatic amino acids enhance DFO-induced cell death. We next assessed whether autophagy was involved in this synergistic effect. Western blot analysis of autophagy markers LC3 and p62 revealed distinct changes following each treatment. In both U87 (Figs. 9C, D) and U251 (Figs. 9E, F) cells, DFO alone induced a robust increase in LC3-II/LC3-I ratio accompanied by a significant decrease in p62 levels, consistent with autophagy-associated changes upon iron chelation. In contrast, supplementation with L-P + L-T in combination with DFO attenuated autophagy-associated changes, as evidenced by the reduction of LC3-II/LC3-I and concurrent accumulation of p62 (Figs. 9C–F). L-P or L-T alone had minimal impact on autophagy changes, emphasizing the necessity for their combined application.

Taken together, these data suggest that DFO induces autophagy-associated responses in GBM cells that may influence the extent of DFO-induced cell death. The addition of L-tyrosine attenuates DFO-induced autophagy-associated marker changes, and was associated with enhanced cytotoxicity. These findings indicate a potential interaction between aromatic amino acid availability and autophagy-related signaling under iron chelation stress. However, the mechanistic relationship and whether autophagy directly mediates the observed sensitization need further investigation.

## Discussion

The present study characterizes the cellular and metabolic consequences of iron deprivation in GBM cells and provides mechanistic insight into how DFO regulate hypoxia signaling, autophagy, apoptosis, ferroptosis resistance, and amino acid metabolic reprogramming. By integrating molecular, functional, and metabolomic analyses via two metabolically distinct GBM models, our findings collectively demonstrate that DFO establishes a complex stress phenotype in which *hypoxia-like* transcriptional activation as well as profound metabolic insufficiency, thereby engaging both adaptive and pro-death pathways.

Firstly, DFO exerted a robust hypoxia-mimetic effect in both U87 and U251 cells, as evidenced by dose-dependent stabilization of HIF-1α and induction of downstream targets. This response was quantitatively more pronounced in U251 cells, which exhibited markedly elevated CA2, CA9, COX-1, and COX-2 expression, suggesting that the transcriptional sensitivity to iron-dependent dioxygenase inhibition differs among GBM subtypes. Importantly, despite the activation of *hypoxia-related* gene expression, DFO simultaneously suppressed cell viability, indicating that HIF-1α stabilization does not rescue GBM cells from the metabolic burden imposed by iron deprivation. Thus, DFO simultaneously triggers *hypoxia-like* transcriptional responses and iron depletion driven metabolic stress, and the interplay between these two processes largely shapes subsequent cell-fate outcomes.

Our data suggest that DFO induces autophagy-associated responses consistent with preserved autophagic flux. The upregulation of ULK1, together with reduced mTOR transcript levels, increased LC3 lipidation together with CQ-dependent LC3-II accumulation support DFO as an autophagy inducer in GBM cells (Kaplan et al. 2008; Wang et al. 2023). However, mTORC1 activity was not directly assessed, such as p-S6K/p-4EBP1 in our study, and involvement of mTORC1 remains a hypothesis. Notably, maintenance of p62 turnover differentiates DFO-induced autophagy from stress conditions that cause autophagosome accumulation due to lysosomal impairment (Victoria Cohen-Kaplana et al. xxxx). The functional relevance of this autophagic response is further supported by the CQ co-treatment. CQ, which blocks lysosomal acidification and prevents autophagosome degradation (Mauthe et al. 2018), significantly potentiated the cytotoxic effect of DFO in both U87 and U251 cells. This enhancement cannot be attributed to CQ toxicity alone, as CQ monotherapy minimally affected viability. Instead, it reflects the loss of an essential compensatory pathway required for cellular survival under iron-deprived conditions. The extent of CQ-mediated sensitization suggests that autophagy functions as a cytoprotective mechanism, attenuating the metabolic and redox perturbations triggered by iron depletion. Therefore, DFO-induced autophagy functions as a cytoprotective response, and its inhibition reveals DFO-mediated lethality.

In parallel, apoptosis contributed to DFO-induced cell death. In U87 cells, DFO downregulated Bcl-2 and modestly increased Caspase-3 activation, indicating apoptotic priming rather than full apoptotic process (Adams and Cory 2017). The absence of significant PARP cleavage further supports the view that apoptosis is not the predominant death mode in U87. By contrast, U251 cells exhibited clear caspase-3 activation in response to DFO, and inhibition of caspase activity by Q-VD-OPh substantially restored cell viability. The differential rescue between the two different GBM cells identified that apoptosis is a major execution pathway in U251 but not in U87. Thus, while DFO establishes upstream apoptotic signaling in both cells, its downstream execution is strongly cell-type dependent. This heterogeneity likely reflects intrinsic differences in mitochondrial reliance, redox tolerance, and basal expression patterns of Bcl-2 family proteins between the two cell lines (Qian et al. 2022).

Furthermore, our ferroptosis-associated marker profile not consistent with ferroptosis under the indicated conditions. Instead, DFO elicited a canonical iron-starvation signature marked by elevated TFR1 expression and preservation of GPX4 protein levels (Alves et al. 2025). The maintenance of GPX4 activity and absence of SLC7A11 suppression are incompatible with ferroptotic commitment, which requires GPX4 depletion or inactivation (Liu et al. 2022). Moreover, the metabolomic detection of enhanced glutathione-related pathways, particularly in U251 cells, further suggests reinforcement of antioxidant defenses rather than susceptibility to lipid peroxidation. Accordingly, these observations are consistent with preserved antioxidant/anti-ferroptotic capacity during DFO-induced iron starvation; however, functional ferroptosis assays will be required to determine whether DFO alters ferroptosis susceptibility.

The metabolomic analyses provide a mechanistic contribution that integrates these cellular features with DFO-induced metabolic remodeling. Both GBM cell lines displayed a highly distinct metabolic reprogramming pattern, with PCA and clustering analyses demonstrating consistent intragroup similarity and pronounced separation from controls. The pathway impact radar plot revealed that phenylalanine/tyrosine/tryptophan metabolism was the most prominently disrupted pathway in both U87 and U251, but the extent of pathway perturbation was substantially greater in U87. This distinction aligns with the global metabolite distribution patterns: U87 displayed a more extensive and pronounced shift in aromatic amino acid metabolism, whereas U251 exhibited a more attenuated metabolic adjustment, despite converging on similar pathway categories (Martins et al. 2023). Beyond the aromatic amino acids highlighted in the radar plot, the two cell lines also exhibited distinct metabolic signatures, reflecting differences in pathway engagement and redox remodeling. U87 cells exhibited more extensive perturbation in central carbon metabolism and broader alterations across multiple amino acid networks, including branched-chain amino acids and arginine-proline pathways, reflecting a more generalized metabolic vulnerability to iron deprivation. In contrast, U251 cells showed pronounced enrichment in glutathione metabolism, suggesting that their adaptation to DFO relies more heavily on reinforcement of antioxidant defenses than on extensive remodeling of amino acid biosynthetic pathways. These cell line-specific metabolic patterns parallel their different dependence on apoptosis and their distinct ability to sustain aromatic amino acid levels under DFO stress, highlighting how metabolic profile influences the stress-response pathways activated by iron chelation.

The functional relevance of these metabolomic signatures was validated by the L-phenylalanine and L-tyrosine supplementation experiments. DFO reduced intracellular aromatic amino acid abundance in U87, consistent with its broader metabolic disruption, whereas U251 exhibited more stable levels. Notably, combined L-P and L–T supplementation attenuated autophagy-associated LC3-II accumulation and altered p62 levels and substantially enhanced DFO cytotoxicity in both lines. These effects mirror the sensitization produced by CQ, indicating that aromatic amino acid availability is critical for maintaining autophagic capacity during iron deprivation (Liu et al. 2021; Srivastava et al. 2025). Integrating the metabolomic profiles with the observed decline in aromatic amino acids and the dependence on autophagy for survival reveals a distinct mechanism: DFO-induced depletion of aromatic amino acids activates autophagy as a protective response, whereas restoring these amino acids through L-P and L–T supplementation suppresses autophagy and increases cellular vulnerability to the metabolic stress imposed by iron loss.

## Conclusion

Our study clarifies the mechanism of DFO action in U87 and U251 GBM cell lines and demonstrates that iron chelation induces a multifactorial stress state, which engages hypoxia signaling, autophagy with cytoprotective features, and variable apoptotic execution while showing a ferroptosis-marker profile not consistent with ferroptotic commitment under these conditions in conjunction with reshaping both amino acid and mitochondrial metabolism. A key insight emerging from this study is the identification of aromatic amino acid metabolism, highlighted by multivariate metabolomics, pathway enrichment, and radar plot analyses, as a modulator of autophagy-associated responses and a key driver of GBM susceptibility to iron deprivation. By combining mechanistic analyses with metabolic profiling, our findings indicate that therapeutic strategies exploiting iron metabolism in GBM cells may be substantially enhanced by co-targeting metabolic adaptation and autophagic survival pathways.

## Supplementary Information

Below is the link to the electronic supplementary material.

Supplementary file1 (PDF 4436 KB)

Supplementary file2 (XLSX 424 KB)

Supplementary file3 (Docx 15,635 KB)

## Data Availability

The datasets used and/or analyzed during the current study are available from the corresponding author on reasonable request. Metabolomic data are stored in data repository “Metabolights” [https:/www.ebi.ac.uk/metabolights] under file number REQ20251212215377.

## Abbreviations

DFO: Deferoxamine
GBM: Glioblastoma
CQ: Chloroquine
LC-3: Light chain
L-P: L-Phenylalanine
L–T: L-Tyrosine
TFR: Transferrin receptor
GPX: Glutathionperoxidase
SLC: Solute Carrier
PARP: Poly (ADP-ribose) polymerase
ULK: Unc-51-like kinase
HIF: Hypoxia-inducible factor
CA: Carbonic anhydrase
COX: Cyclooxygenase
ATG: Autophagy
mTOR: Mechanistic target of rapamycin
BECN: Beclin
AMPK: AMP activated kinase
SQSTM: Sequestosom
