# Supplementary figures and images for: Aromatic amino acid metabolism shapes autophagy-mediated adaptation to iron deprivation in glioblastoma cells
Source: Biometals. 2026 Apr 2;39(3):1167–89. doi: 10.1007/s10534-026-00809-7 (PMC13230266; doi:10.1007/s10534-026-00809-7)

Fig 1A

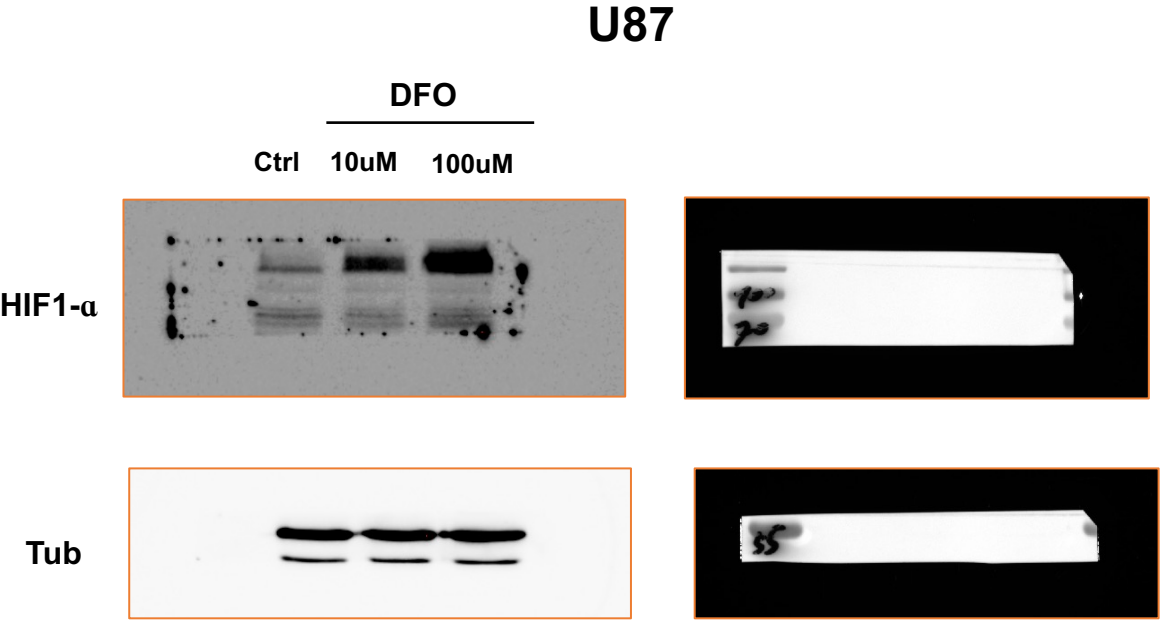

Fig 1B

U251

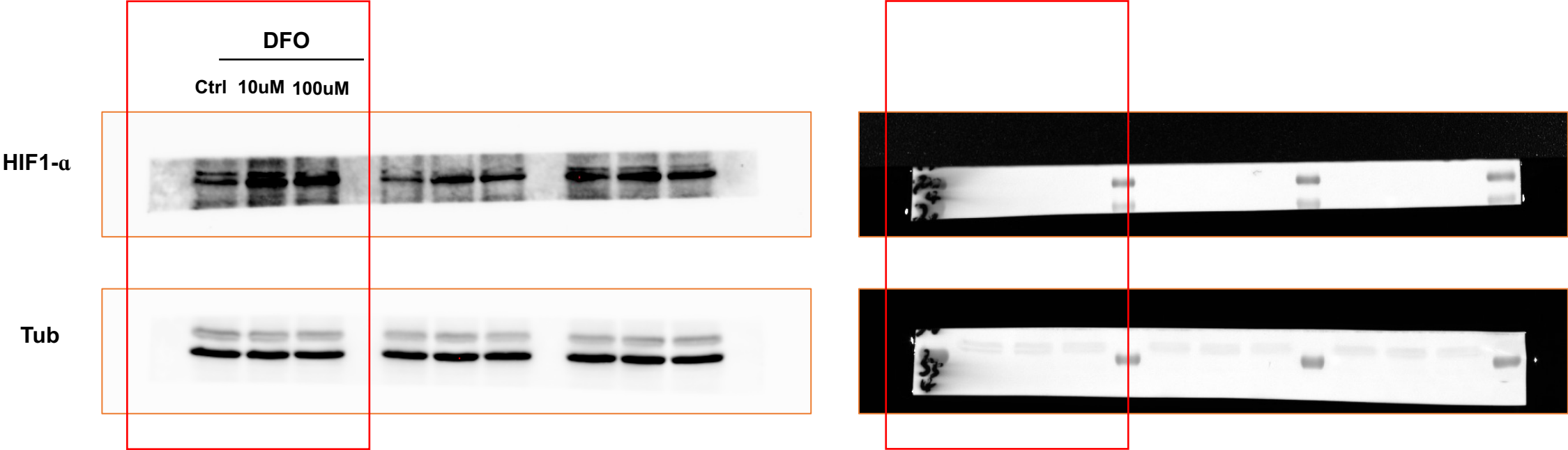

Fig 2D

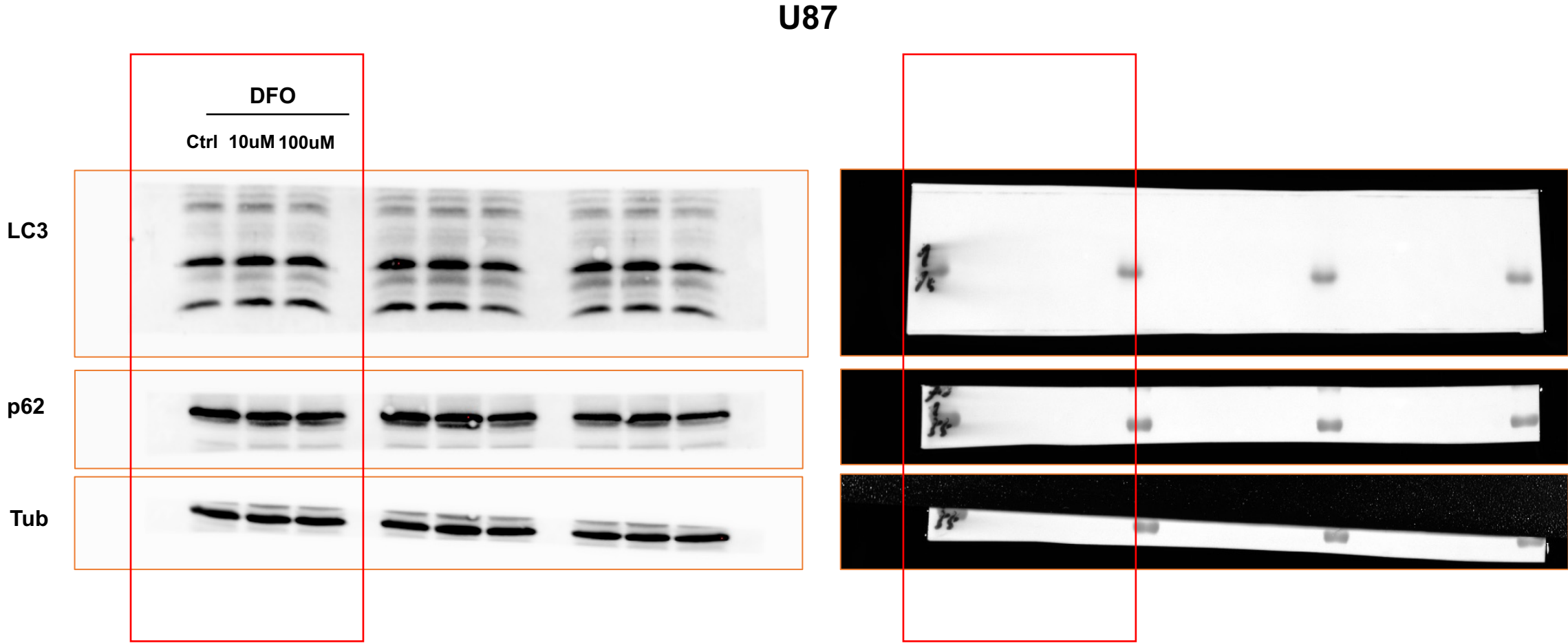

Fig 2F

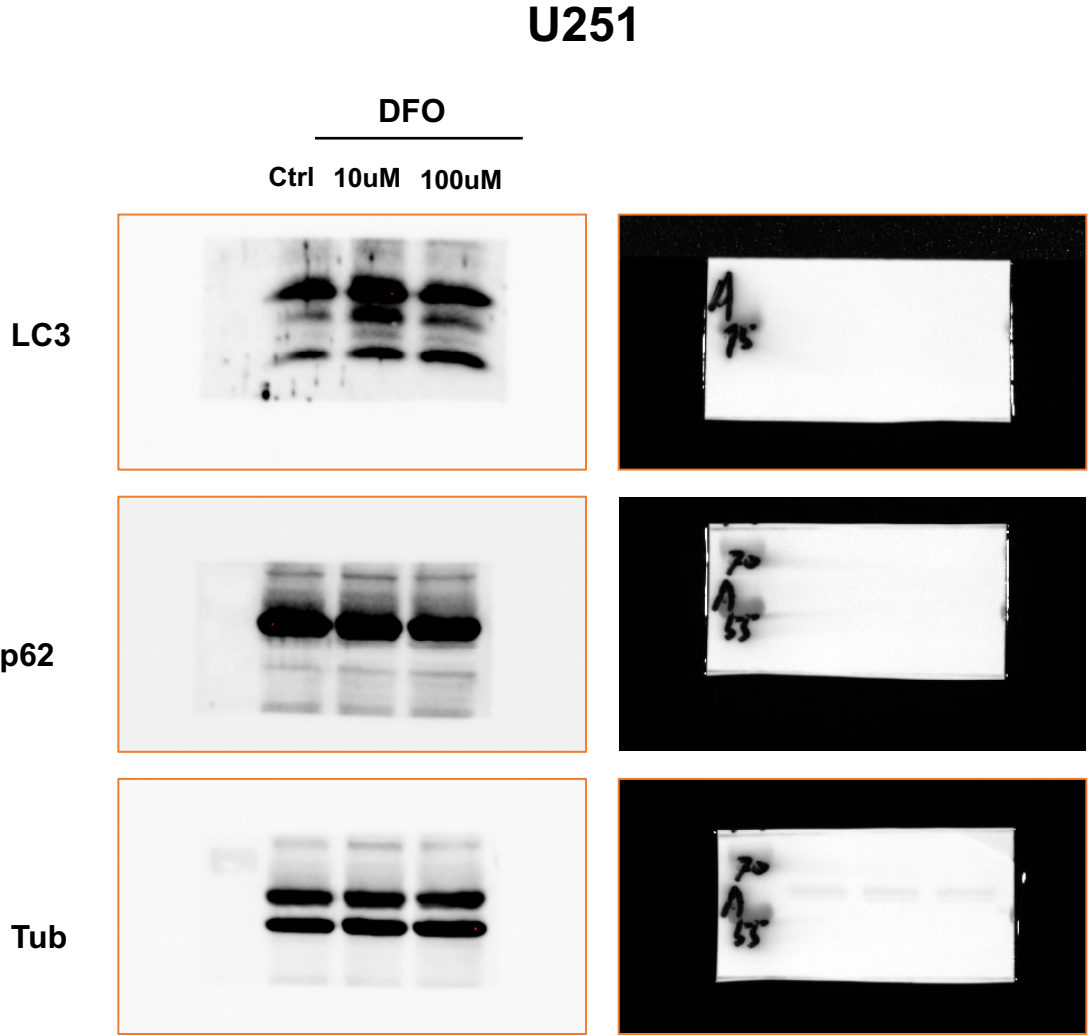

Fig 3D

U87

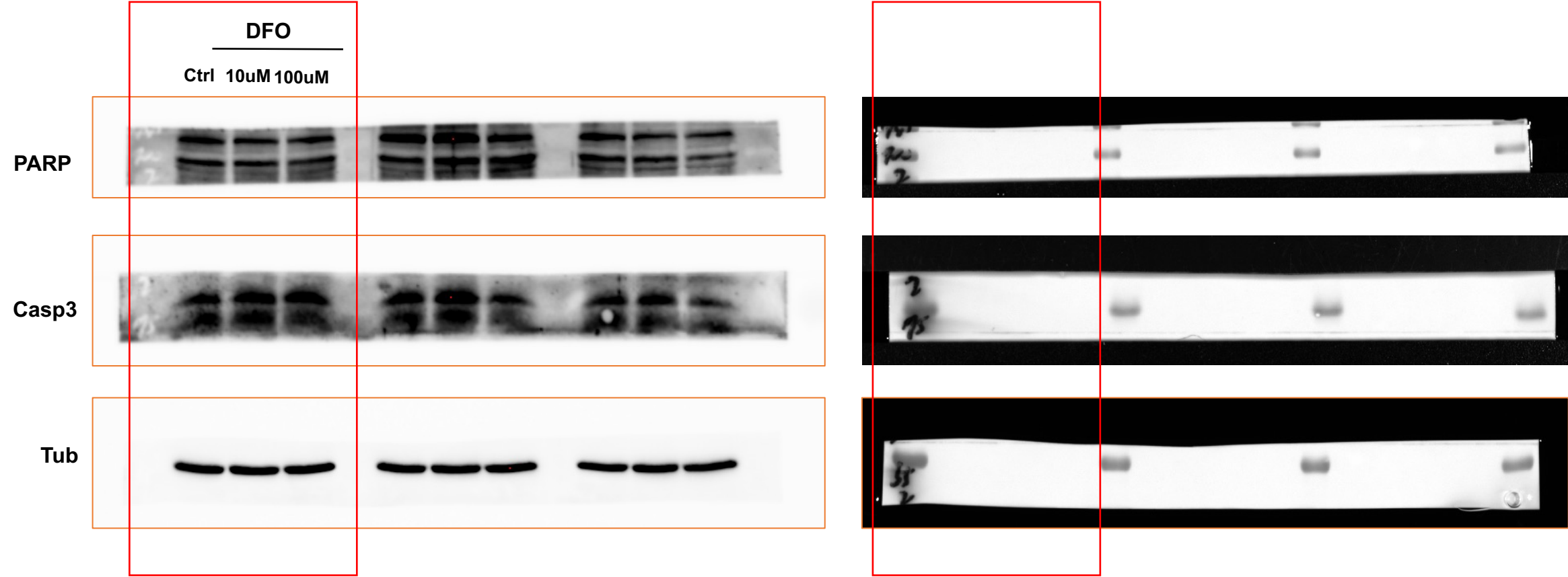

Fig 3F

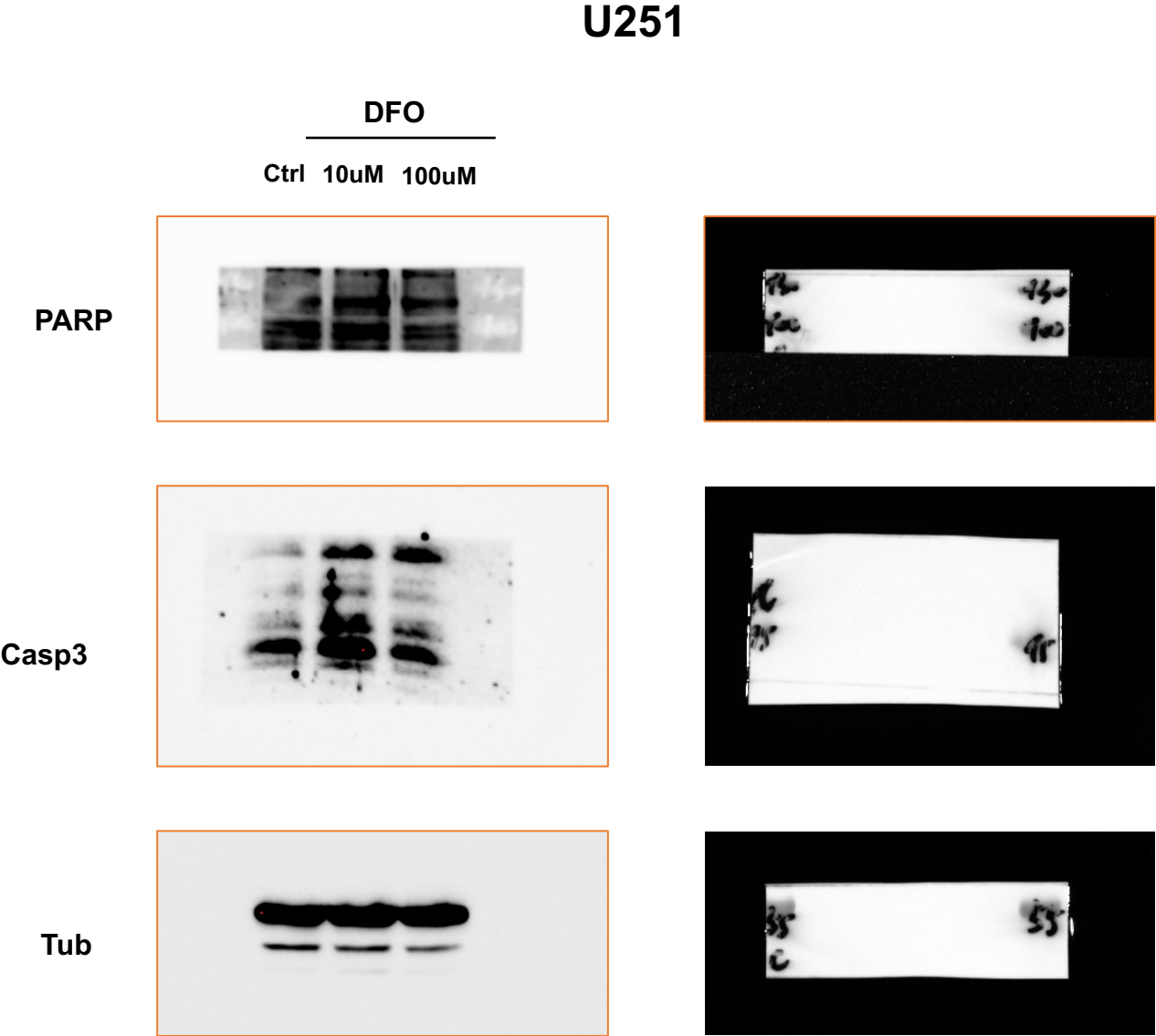

Fig 4D

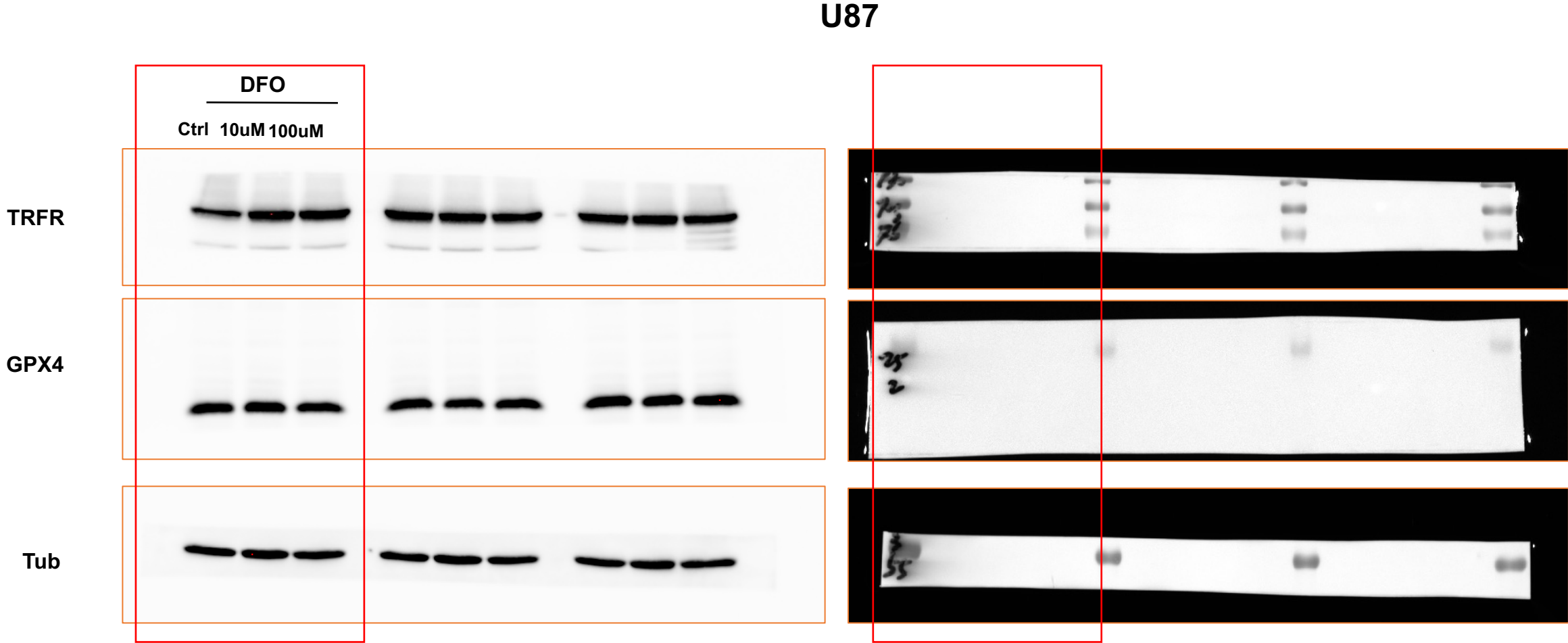

Fig 4F

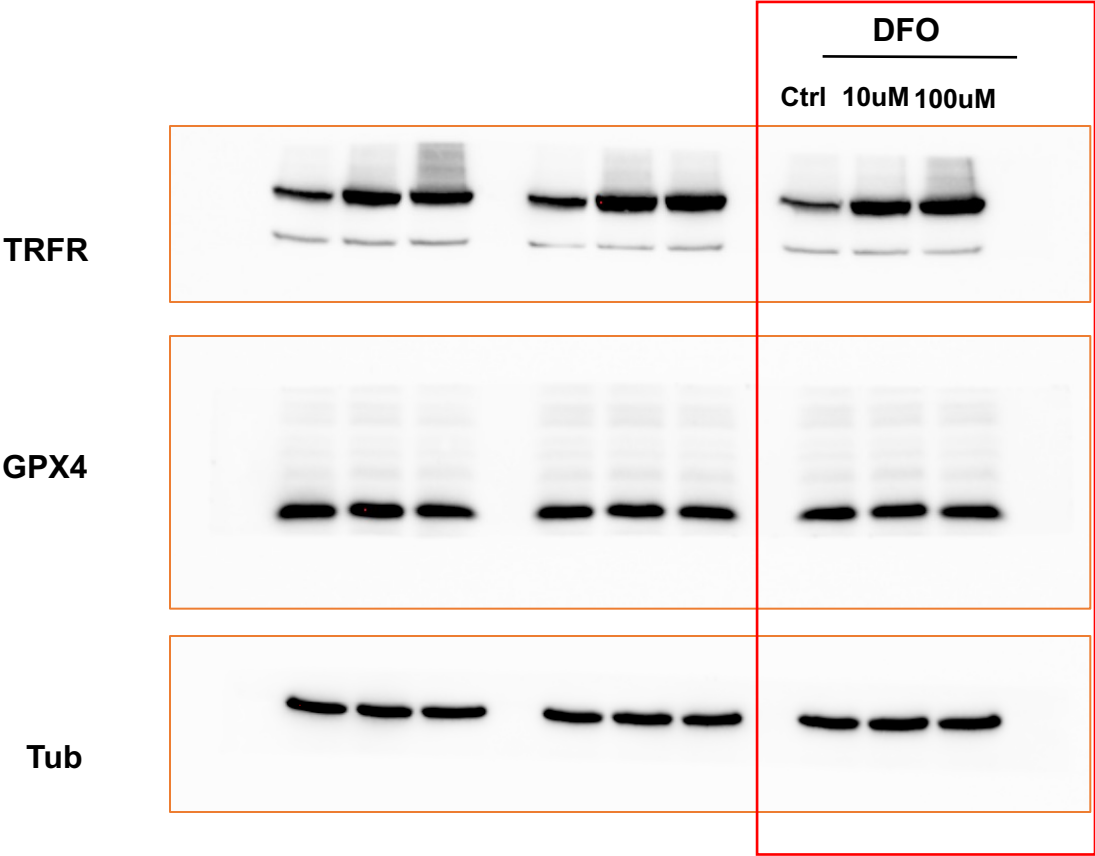

U251

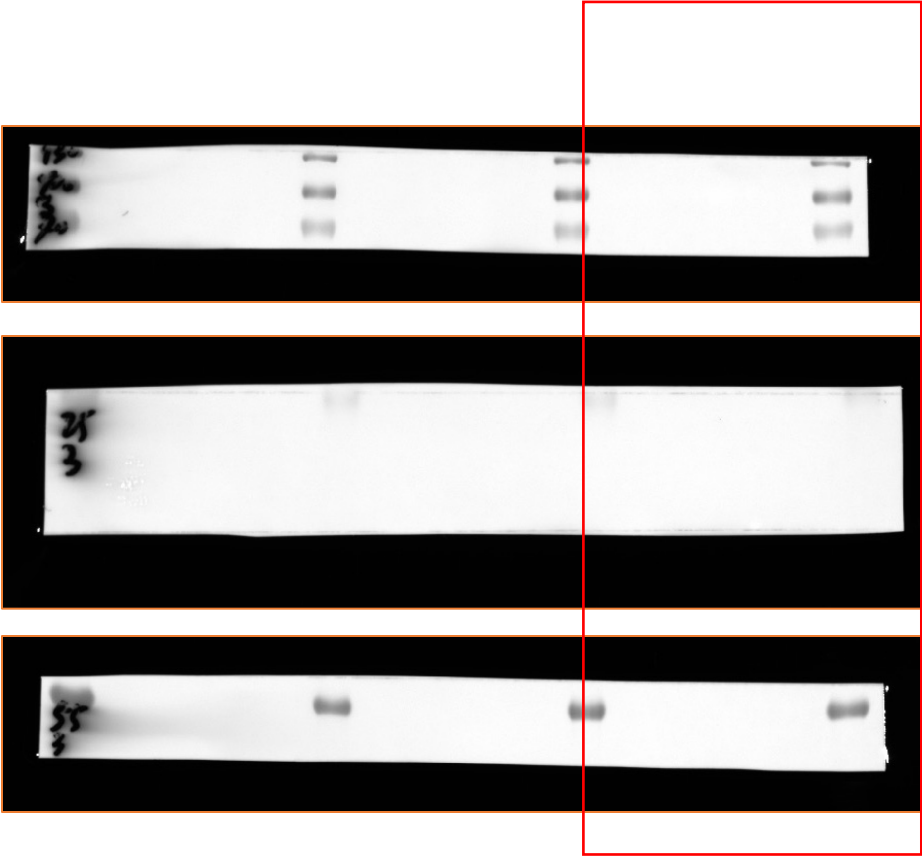

**Fig 9C**

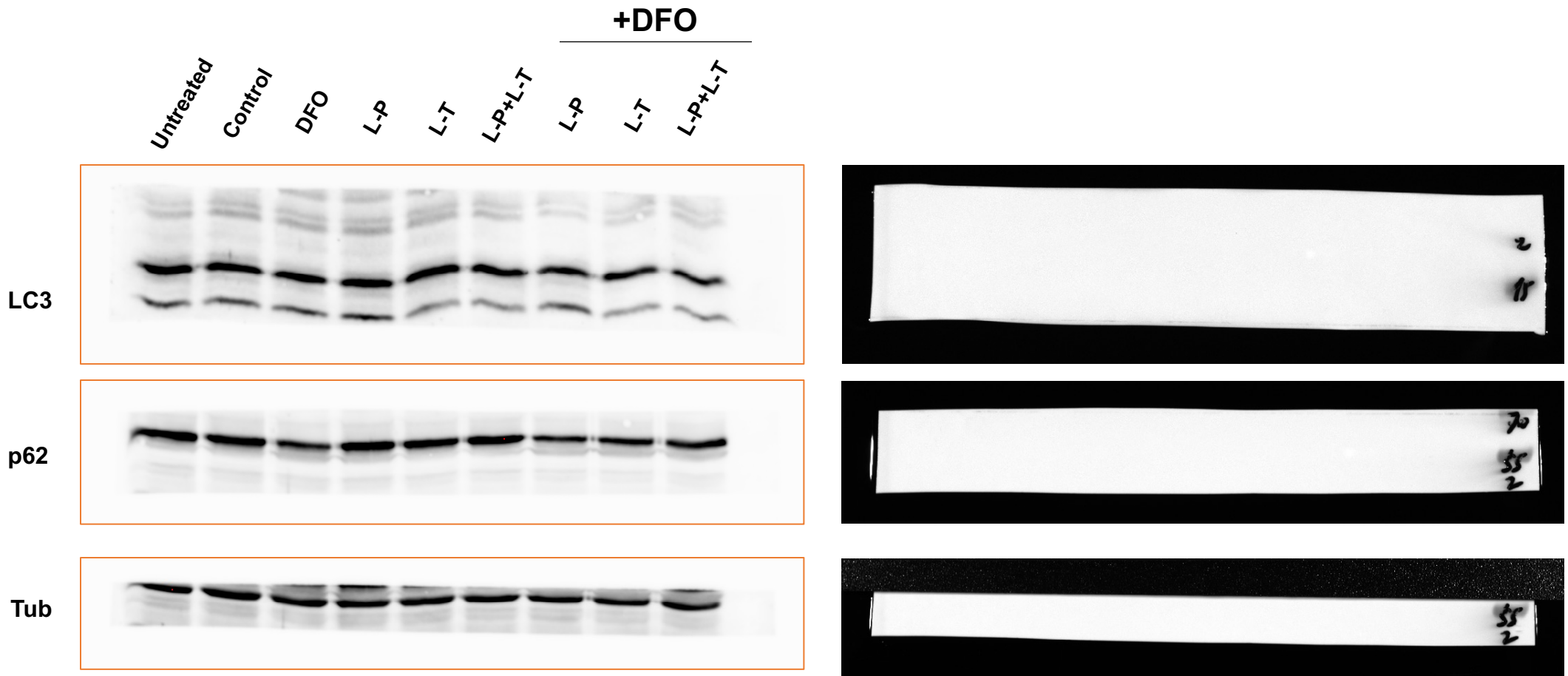

**Fig 9D**

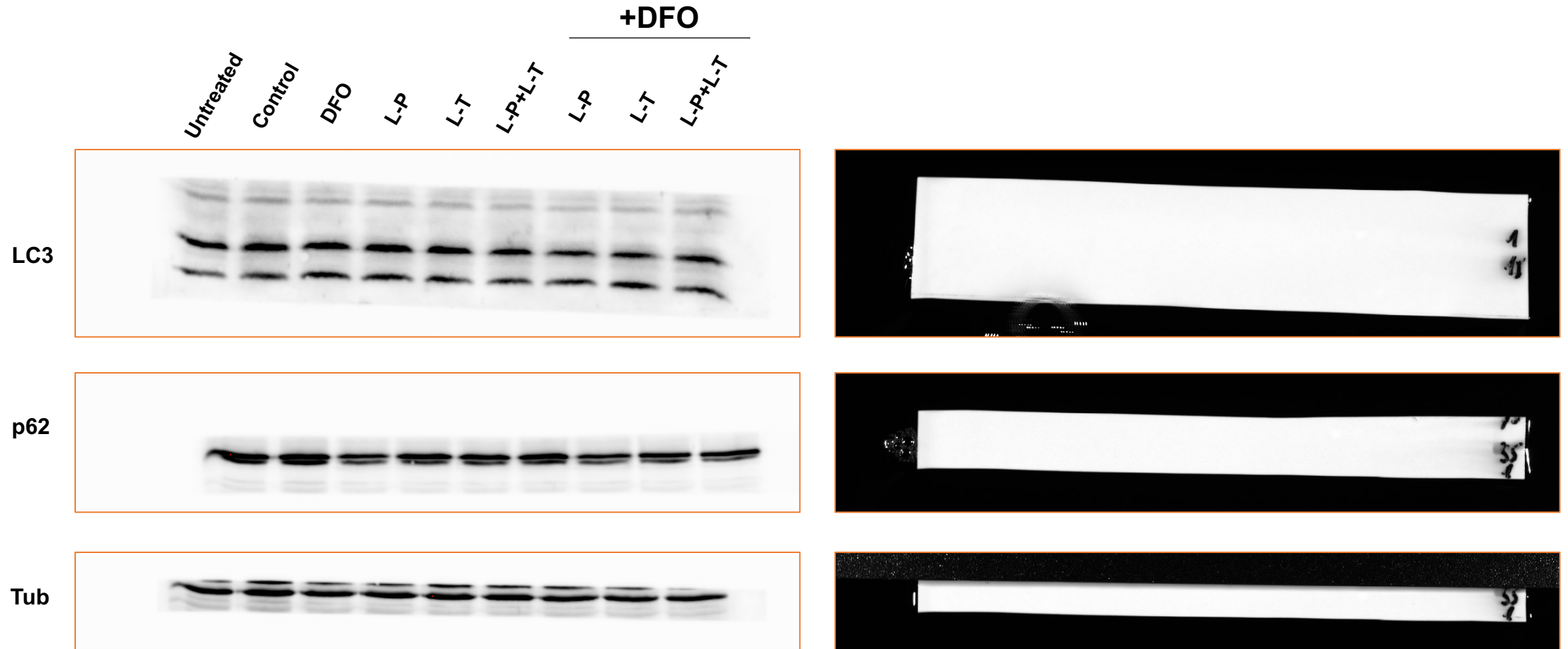

Supplement: Supplementary file 1 — Supplementary file1 (PDF 4436 KB) [file 10534_2026_809_MOESM1_ESM.pdf]
