# Supplementary material for: Aromatic amino acid metabolism shapes autophagy-mediated adaptation to iron deprivation in glioblastoma cells
Source: Biometals. 2026 Apr 2;39(3):1167–89. doi: 10.1007/s10534-026-00809-7 (PMC13230266; doi:10.1007/s10534-026-00809-7)
Supplement: Supplementary file 3 — Supplementary file3 (Docx 15,635 KB) [file 10534_2026_809_MOESM3_ESM.docx]

**
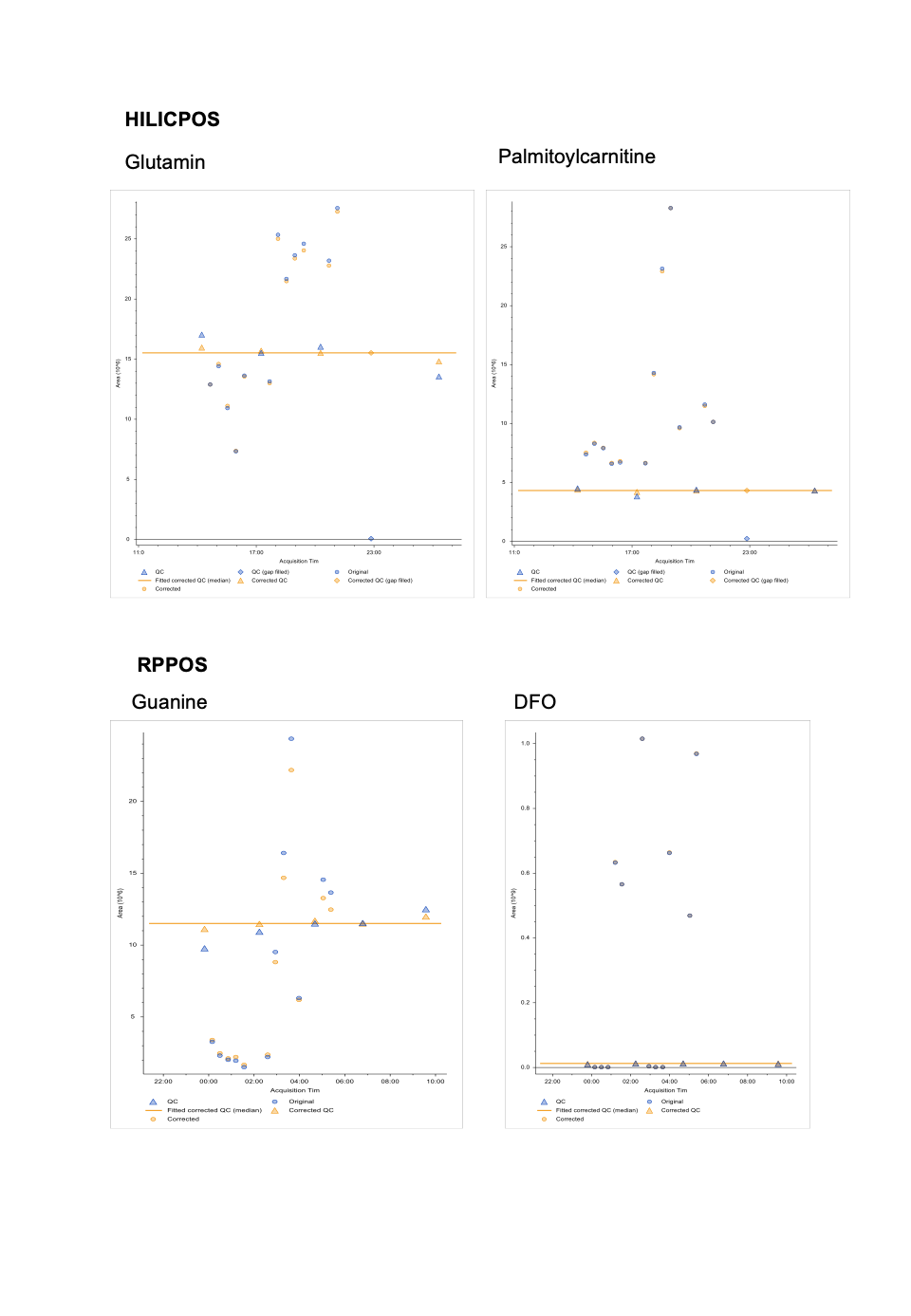
**

**
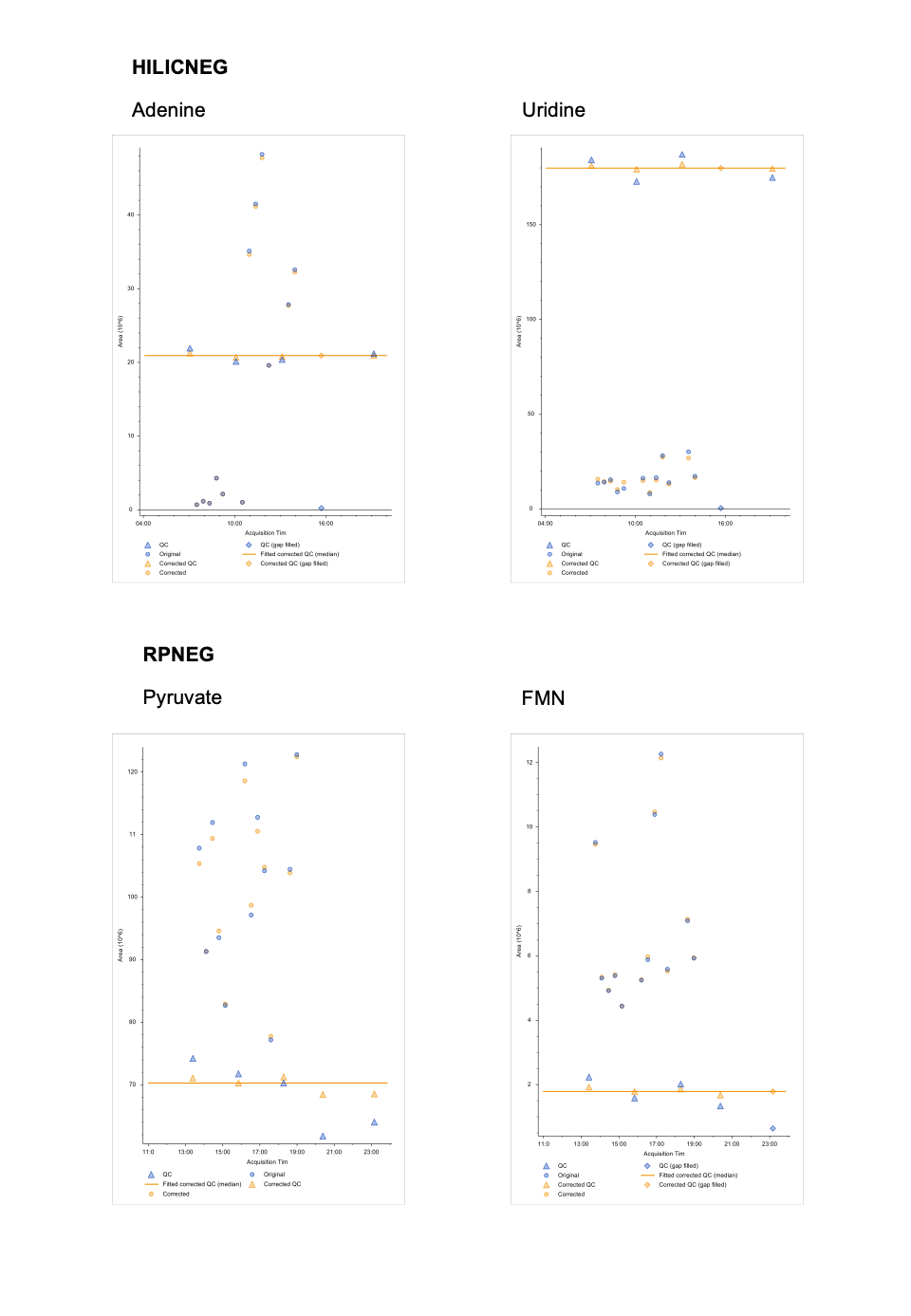
**

**Supplementary Figure S1.** **QC correction and representative LC–MS chromatograms.**

Representative extracted ion chromatograms from HILIC positive (HILICPOS), reverse-phase positive (RPPOS), HILIC negative (HILICNEG), and reverse-phase negative (RPNEG) modes are shown. Example metabolites include Glutamine, Palmitoylcarnitine, Guanine, DFO, Adenine, Uridine, Pyruvate, and FMN. QC-based signal correction was applied prior to analysis, and representative peaks demonstrate stable retention times and consistent signal quality.

**
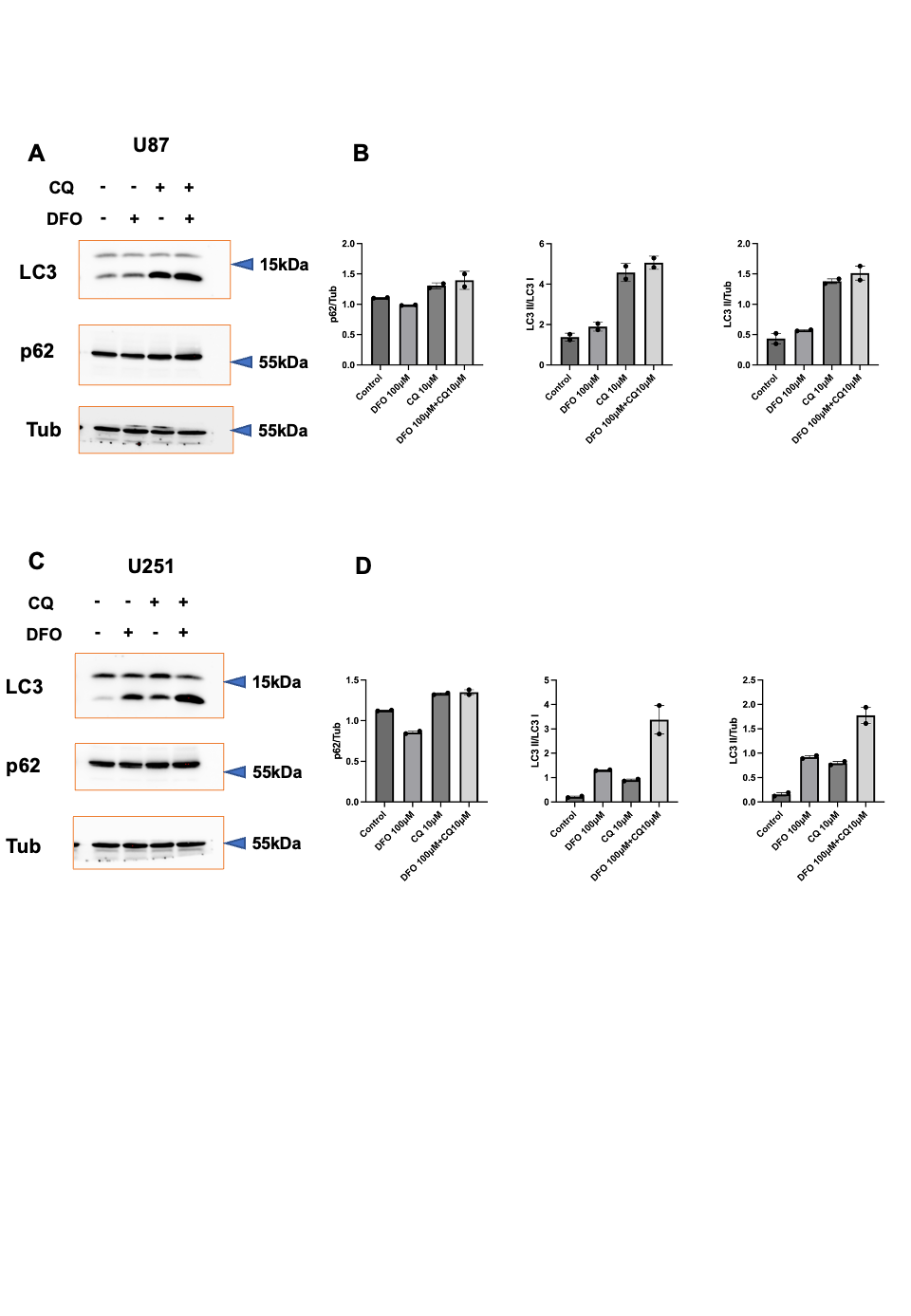
**

**Supplementary Figure S2.** **CQ-dependent LC3-II accumulation in DFO-treated GBM cells.**

(A, C) Representative western blots of LC3, p62, and tubulin in U87 (A) and U251 (C) cells treated with DFO (100 μM, 24 h) in the presence or absence of chloroquine (CQ, 10 μM).

(B, D) Densitometric quantification of p62/Tub, LC3-II/LC3-I, and LC3-II/Tub ratios. Data are presented as mean ± SME from two independent experiments. CQ co-treatment resulted in further LC3-II accumulation compared with DFO alone.

**
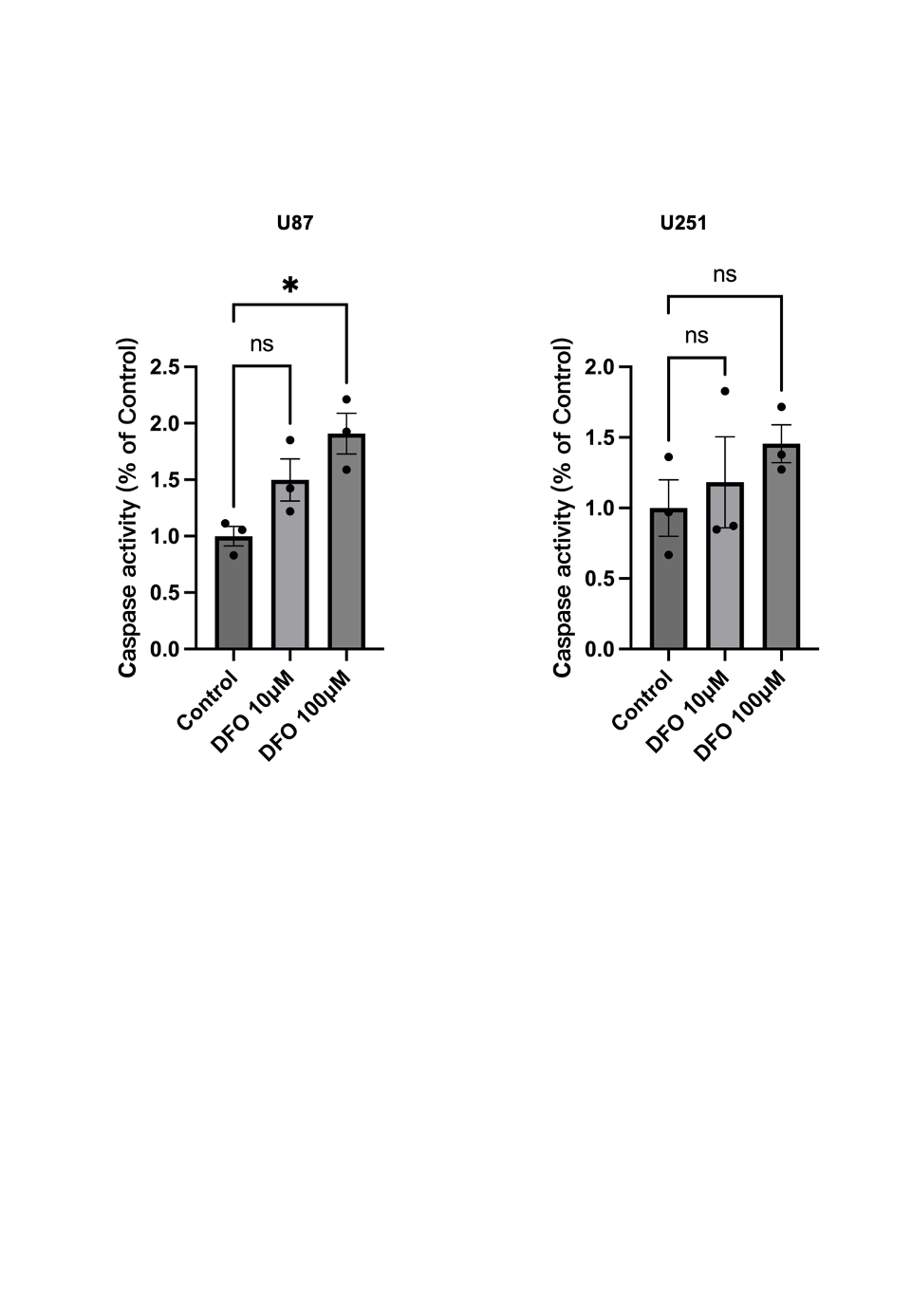
**

**Supplementary Figure S3. Caspase activity in DFO-treated GBM cells.**

Caspase activity was measured using the Caspase-Glo assay in U87 and U251 cells treated with DFO (10 μM or 100 μM) for 24 h. Data are expressed as percentage of control and presented as mean ± SD from independent experiments. Statistical significance was determined by one-way ANOVA (*p < 0.05; ns, not significant).
